# Comparison of Pesticide Residue Levels in Red Wines from Canary Islands, Iberian Peninsula, and Cape Verde

**DOI:** 10.3390/foods9111555

**Published:** 2020-10-27

**Authors:** Álvaro Santana-Mayor, Ruth Rodríguez-Ramos, Bárbara Socas-Rodríguez, Carlos Díaz-Romero, Miguel Ángel Rodríguez-Delgado

**Affiliations:** 1 Departamento de Química, Unidad Departamental de Química Analítica, Facultad de Ciencias, Universidad de La Laguna (ULL), Avenida Astrofísico Francisco Sánchez, s/n. 38206 San Cristóbal de La Laguna, Tenerife, Spain; asantanm@ull.edu.es (Á.S.-M.); rrodrira@ull.edu.es (R.R.-R.); 2 Laboratory of Foodomics, Institute of Food Science Research, CIAL, CSIC, Nicolás Cabrera 9, 28049 Madrid, Spain; 3 Departamento de Ingeniería Química y Tecnología Farmacéutica, Unidad Departamental de Nutrición y Bromatología, Facultad de Ciencias, Universidad de La Laguna (ULL), Avenida Astrofísico Francisco Sánchez, s/n. 38206 San Cristóbal de La Laguna, Tenerife, Spain; cdiaz@ull.edu.es

**Keywords:** organic contaminants, alcoholic beverages, pesticide occurrence, agricultural contamination, Macaronesia, Spain

## Abstract

In this work, the QuEChERS method coupled to liquid chromatography-time-of flight-mass spectrometry and gas chromatography-triple quadrupole-mass spectrometry were applied for the evaluation of pesticide residues and risk assessment in red wines. The methodologies were successfully validated for 173 pesticides. Recovery values were in the range 75–100% for almost all pesticides and limits of quantification were between 2.60 and 21.39 µg/kg, which are in good agreement with the maximum residue limits (MRLs) established by the European Commission for pesticides in wine grapes. Finally, the analysis of 84 red wine samples from the Canary Islands, the Iberian Peninsula, and Cape Verde was carried out, which found the presence of 31 pesticide residues. However, the risk assessment disclosed that despite the large number of pesticides and the concentrations found, which in some cases exceeded the MRLs, the consumption of these wines, without considering a possible cumulative effect, does not entail a risk to the consumers.

## 1. Introduction

In recent years, human beings have been submitted to a wide variety of harmful agents, largely due to the quick evolution of society. Among them, pesticides have been one of the most widely used and have caused great damage to people due to their known toxicity. These compounds are increasingly relevant in the growth of developing countries. This may lead to an increase in the use of pesticides as a means of crop protection due to general population growth and the consequent need for increasing agricultural production [1]. All this has highlighted a potential risk for these countries, both for human health and for the environment. Just as pesticides are beneficial for agricultural production and for protecting crops from pests or preserving the derived products, the harmful effects on health that can causethe consumption of food contaminated with these compounds, and its subsequent bioaccumulation represent a risk for mammals [1,2]. Moreover, certain plant protection products of synthetic origin used in agricultural labours are persistent in the environment. In this way, through the food chain and the contact with the environment, society is exposed to a great mixture of pesticides, which can entail greater danger to health [1].

The search for new systems that allow modifying current practices towards a more ecological agriculture, and reducing or eliminating the use of pesticides, is essential. In this way, it would ensure the preservation of ecosystems, as well as the protection of citizens and, above all, of especially vulnerable groups [3].

Organochlorine pesticides (OCPs) are persistent pollutants that have been linked with several deleterious effects on human health [4], including breast cancer [5]. In addition, several OCPs and some of their metabolites are considered endocrine disruptors and have been found to induce oestrogen-like effects in humans [6]. In fact, human exposure to persistent organic compounds and its consequences have been extensively studied. In contrast, non-persistent pesticides have hardly been evaluated, considering they are chemically unstable compounds that do not accumulate in the body. However, their potential association with developmental, neurocognitive, or endocrine pathologies, and their carcinogenic and mutagenic effects [7], also make it necessary to know the degrees of exposure to non-persistent pesticide residues.

In the Canary Islands, extensive farming areas have been developed in recent decades. It is the Spanish region with the highest expenditure on phytosanitary products; there is a consumption of 69.1 kg/ha, which is almost 20 times higher than the Spanish average of 3.6 kg/ha, while the area dedicated to organic crop farming, in relation to the useful agricultural area, is only 3.6% [8]. Apart from that, the incidence of cancer in this region, including breast and bladder cancer, has increased since the 1950s. The level of contamination by environmental chemicals and its potential role as a risk factor for cancer on the population of the Canary Islands have been extensively studied [9,10]. In one of those studies [9], carried out in 363 serum samples obtained from non-occupationally exposed adults from Tenerife island, 99.4% of the individuals had non-persistent pesticide residues in their bodies, with an average of six different pesticides per person. Bifenthrin and malathion were the most detected residues. There is an inadvertent exposure to non-persistent pesticides that can affect population health, making it necessary to include them in more exhaustive and strict monitoring studies.

Wine constitutes a highly consumed product among the Canarian and Spanish population. However, the European Union (EU) has not established limits of pesticide residues in wine yet. In its absence, those settled for vinification grapes are generally accepted for wine. In this regard, the latest data collected in Commission Implementing Regulation (EU) 2018/555 [11] includes useful specific information about transformation factors of raw materials into transformed products for future regulations. Regarding the list of pesticides authorised in vineyards and their maximum residue limits (MRLs) in wine grapes, these vary depending on the producing countries [12]. That is why, it is necessary to develop analytical methods that provide high sample throughput, moderate costs, and limits of quantification (LOQs) low enough to fulfil the different regulations of each country. Besides, the increased demand of ecological wines is fostering the development of ultra-sensitive methods capable of guaranteeing the standards of quality.

In this regard, the presence of pesticide residues in red wines from the Canary Islands has only been assessed in the work developed by Ravelo-Pérez et al. in 2008 [13]. Regarding the analysis of pesticides in this matrix from other regions of Spain, five studies have been reported in the last decade [14,15,16,17,18]. Three of them, developed by the same research group, have carried out the analysis of red wine samples from the Northwest of Spain [14,15,16]. Likewise, Pérez-Ortega et al. [17], performed the analysis of 24 samples from different regions of Spain, and found the presence of nine pesticide residues belonging to fungicide and insecticide families in some of them. In the study developed by Romero-González et al. [18], pesticides were analysed in wine samples, among other organic food products, from the South of Spain. Up to know, it has not been any data reported in the literature regarding the occurrence of the pesticide content in wines produced in Cape Verde; thus, the information reported in this work can serve as a reference for other studies.

In this paper, the content of pesticide residues in red wine samples produced in the Canary Islands and other Spanish regions, as well as Cape Verde, was determined for the first time for the evaluation of the exposure of the population to this kind of environmental contaminants. This paper also, for the first time, compares the concentration levels found in each area with the values permitted by the established regulations. With this aim, the QuEChERS (Quick, Easy, Cheap, Effective, Rugged, and Safe) method combined with ultra-high performance liquid chromatography (UHPLC) and gas chromatography (GC) coupled to tandem mass spectrometry (MS/MS) was applied for the analysis of a wide and representative selection of commonly used pesticides belonging to several families and with different chemical activities. Thus, this study has significantly increased the dataset of pesticides occurence in red wines produced in the studied regions.

## 2. Materials and Methods

### 2.1. Chemicals and Reagents

Analytical standards of pesticides, all of them with purity higher than 95.9%, were obtained from Sigma-Aldrich (St. Louis, MO, USA) and Dr. Ehrenstorfer GmbH (Augsburg, Germany). Names and data related to the 59 analytes determined by liquid chromatography (LC)-MS/MS and the 114 analysed by GC-MS/MS are listed in Tables S1 and S2, respectively. Triphenylphosphate (TPP), purity 99.7%, which was used as a surrogate and as quality control, was purchased from Dr. Ehrenstorfer GmbH (Augsburg, Germany).

Individual stock solutions of each analyte were prepared in acetone at 1000 mg/L and mixed solutions were also prepared in this solvent at 10 mg/L and stored in darkness at −18 °C. Working standard mixtures of all pesticides were daily prepared by dilution with an appropriate volume of cyclohexane/ethyl acetate (EtOAc) (9/1, *v*/*v*).

All chemicals were of analytical reagent grade (unless otherwise indicated) and used as received. Acetonitrile (ACN) and methanol (MeOH) of LC-mass spectrometry (MS) grade, and cyclohexane of GC-MS grade were from Merck (Darmstadt, Germany). EtOAc of high performance liquid chromatography (HPLC) grade was from Sigma-Aldrich Chemie (Madrid, Spain). Ammonium acetate was from Scharlau Chemie S.A. (Barcelona, Spain). QuEChERS extraction kits (containing MgSO_4_, NaCl, trisodium citrate dihydrate and disodium hydrogencitrate sesquihydrate) and QuEChERS dispersive kit (containing primary secondary amine (PSA), octadecylsilane (C_18_) sorbent and MgSO_4_) were from Agilent Technologies (Waldbronn, Germany). Water was deionised by Milli-Q gradient system A10 from Millipore (Burlington, MA, USA).

### 2.2. QuEChERS Extraction Procedure

The QuEChERS method was applied following the European Standard EN 15662 [19] for the multiresidue analysis of pesticides from wines with different origin and nature. Briefly, 10.0 ± 0.1 g of wine sample was weighed into a 50 mL centrifuge tube, 100 µL of surrogate solution of 10 mg/L and 10 mL of ACN were added. Then, the tube was manually shaken for 1 min. Subsequently, the mixture was transferred to a QuEChERS extraction tube, containing 4 g of MgSO_4_ anhydrous, 1 g of NaCl, 1 g of trisodium citrate dihydrate and 0.5 g of disodium hydrogencitrate sesquihydrate, and two ceramic homogenisers; it was shaken again for 1 min and centrifuged for 5 min at 3220× *g* in a 5810 R centrifuge from Eppendorf (Hamburg, Germany). For clean-up step, 6 mL of the supernatant was transferred into a 15 mL polypropylene centrifuge tube containing 150 mg of PSA, 150 mg of C_18_, and 900 mg of MgSO_4_, vigorously shaken for 1 min, and centrifuged for 5 min at 3220× *g*. Afterwards, 500 µL of the supernatant was collected and dried under a gentled nitrogen stream at 40 °C in a 24 position N-EVAP nitrogen evaporator from Organomation Associates, Inc. (Berlin, MA, USA). Finally, the residue was re-dissolved by 500 µL of cyclohexane/EtOAc mixture (90/10, *v*/*v*) prior to GC analysis, or MeOH/water mixture (50/50, *v*/*v*) with 10 mM in ammonium acetate for LC analysis, filtered through a 0.20 µm polytetrafluoroethylene membrane syringe filter and injected into the chromatographic system.

### 2.3. UHPLC-MS/MS Analysis

For LC-MS/MS analysis, a Waters Acquity UPLC^®^ I-Class (Milford, MA, USA), constituted by a binary solvent manager and a sample manager with a flow-through needle and controlled with the Masslynx^TM^ V4.1 software from Waters Chromatography, was used. The UHPLC system was coupled to an MS Xevo^®^ G2-XS quadrupole time-of-flight (Q-ToF) detector (Waters Chromatography) with an electrospray ionisation interface working in positive mode. Control of MS parameters and collection and processing of spectrum data were carried out using the same software from Waters Chromatography. Separation was developed in an Acquity UPLC^®^ BEH C_18_ column (100 × 2.1 mm, 1.7 µm) using an Acquity UPLC^®^ BEH C_18_ pre-column (5 × 2.1 mm, 1.7 µm), both from Waters Chromatography. Column and pre-column temperature were settled at 45 °C.

The mobile phase used for the analysis consisted of MeOH (A) and water (B), both containing 10 mM of ammonium acetate. The initial composition of the mobile phase consisted of 2/98 (*v*/*v*) A/B with a flow rate of 0.45 mL/min. It was changed to 99/1 (*v*/*v*) A/B in 12.25 min, which was maintained during 1.75 min. Finally, the initial conditions were set up in 0.5 min and maintained during 2.5 min. The injection volume was 10 µL at 10 °C.

The MS system was operated in MS^E^ data independent acquisition mode. Retention time, one exact-mass precursor and, at least, 1 fragment ion with a maximum tolerance of ±30% for the relative ion intensities, were set as identification points [20]. Source conditions were as follows: capillary voltage 1.0 kV, source temperature 120 °C, desolvation temperature 500 °C, cone gas (N_2_) flow 20 L/h, desolvation gas (N_2_) flow 1000 L/h, collision gas (Ar) pressure 0.5 bar. The MS was operated in full scan ToF-MS mode (m/z 50–1200 Da). Collision energy was settled at 4 V and 10–45 V for low and high energies, respectively, and the cone voltage was 40 V.

### 2.4. GC-MS/MS Analysis

For GC-MS/MS analysis, an Agilent 7890B GC system coupled to a 7000C triple quadrupole (QqQ) mass spectrometer with an electron impact interface (Agilent Technologies, Waldbronn, Germany) was employed. The GC system was equipped with a GC Sampler 80 and controlled with the GCQQQ/Enhanced MassHunter Software from Agilent Technologies. The separation of the target analytes was carried out in two capillary columns of (5% phenyl)-methylpolysiloxane fused silica (HP-5ms; 15 × 0.25 mm, 0.25 μm film thickness, Agilent Technologies) connected by a backflush system. Helium, as the carrier gas, was set at a flow of 1.0 and 1.2 mL/min in the first and second columns, respectively.

Column temperature was initially set at 60 °C and maintained for 1 min. Then, it was changed to 170 °C at a rate of 40 °C/min, increased to 310 °C at a rate of 10 °C/min and held for 3 min. The run time was 20.75 min. The injection volume was 2 µL in splitless mode at 280 °C.

The MS system was operated in multiple reaction monitoring mode, considering as identification points 1 precursor and, at least, 2 products ions, as well as the retention time for each target analyte and establishing a maximum tolerance of ±30% for the relative ion intensities of the product and precursor ions [20]. Mass spectrometer conditions were as follows: the transfer line and the ion source temperatures were set at 280 °C with an ionisation energy of −70 eV, the quadrupoles temperatures were both set at 180 °C, and the flows of nitrogen and helium, as the collision and quenching gases, were 1.5 and 2.25 mL/min, respectively.

### 2.5. Statistical Analysis

IBM SPSS Statistic (SPSS Inc., Chicago, IL, USA) and Microsoft Excel (Microsoft Office, Redmond, WA, USA) were used for all statistical analyses. All the statistics were performed by means of the SPSS version 17.0 software for Windows (SPSS Inc., Chicago, IL, USA). Mean values obtained for the variables studied in the different groups were compared by nonparametric Mann-Whitney U test (non-normal statistical distribution). There are significant differences among the mean values if the statistical *p*-value is <0.05.

### 2.6. Wine Samples

Eighty-four commercial red grape wines were analysed from three regions. Forty-eight red wine samples were elaborated by the main wine-producers from Canary Islands; thirty samples were produced in wineries from the mainland in Spain and six samples came from Cape Verde. Wine samples were collected between 2017 and 2019 and they belonged to several vintages between 2012 and 2017, except one of them that was produced in 2001. All wines were conventionally produced, except five of them, which had the label of ecological products. The information related to the analysed samples (type of wine, and geographic origin) is shown in Table S3. Samples which were analysed in a 5-day interval were stored in the refrigerator at 4 °C, while those that were analysed in a longer period were divided in aliquots of 10 g and stored in an ultra-freezer at −80 °C.

## 3. Results and Discussion

### 3.1. Validation of Analytical Methods for Pesticide Residues

In this study, a QuEChERS-based procedure according to European Standard EN 15662, prior to analysis by UHPLC-(Q-ToF)-MS/MS or GC-QqQ-MS/MS, was developed for determining a wide group of pesticides in red wines. These pesticides (173 compounds) were selected based on their use as active substances by grape growers in these regions and covering different classes of analytes usually determined by laboratories worldwide.

Method validation was initially conducted for both procedures in order to assess their suitability for the evaluation of the selected compounds, as well as to assure the reliability of the obtained results. With this aim, the following parameters were evaluated: linearity, trueness (recovery), precision (relative standard deviation (RSD) intra-day) and sensitivity (LOQ).

Table 1 compiles the figures of merit obtained for those pesticides that were detected in at least one red wine sample. However, a total of 173 pesticides (59 determined by LC-MS and 114 by GC-MS) were analysed and the analytical information of all of them was included as Supplementary Material (Tables S1 and S2, Figures S1 and S2).

The matrix effect was evaluated by comparison of solvent and matrix-matched standards. The results showed the existence of the matrix effect for most compounds. For this reason, linearity was evaluated for both techniques by the preparation of individual matrix-matched calibration curves using an ecological red wine that was previously analysed, in order to check the absence of pesticides. Seven different concentration levels within the interval of 10–150 μg/L for UHPLC-MS/MS and 10–200 μg/L for GC-MS/MS (in the final extract) were injected, except for the pesticides indicated in the footnote of Table S1, for which were 2–150 μg/L for carbofuran, 15–150 μg/L for flutriafol, iprovalicarb, mepanipyrim, and carbaryl, 10–100 μg/L for bromuconazole, hexaconazole, triasulfuron, and tribenuron, and 20–150 µg/L for thiamethoxam. The determination coefficient (R^2^) values for all the pesticides were clearly above the minimum of 0.9901 indicating good linearity in the intervals considered.

For recovery study in the LC-MS system, five different replicates of ecological red wine were spiked at the beginning and at the end of the procedure at a concentration of 75 g/kg for each pesticide, submitted to the developed QuEChERS-UHPLC-(Q-ToF)-MS/MS method, and their areas were compared. Recovery values varied between 61 and 108%, which are acceptable results based on the validation parameters established by SANTE Guidance [20] for routine analysis. Most of them (46), 78% of the pesticides analysed by this procedure, showed good recovery (based on the same guidance), in the range 75–100%, which includes the 11 pesticides that have been detected in at least one red wine sample using this technique (Table 1). For the GC-QqQ-MS/MS method, a recovery study was performed at two concentration levels (10 µg/kg—near the LOQ; and 100 µg/kg—near to 10 times the LOQ) following the same procedure as in LC. Five red wine aliquots were spiked for each concentration assayed. For most of compounds (99), 86.8% of the pesticides analysed with this technique, good recovery values, in the range 75–100%, were obtained, except for only three of them (i.e., endosulfan sulphate, chlorothalonil and quinomethionate). The 20 pesticides that were detected in at least one sample of the red wine analysed by this technique had a recovery within that range (Table 1). RSD values ranged between 5% and 20% for both techniques, which demonstrates good performance of the developed methodologies in terms of precision and trueness. The great majority of pesticides studied, 52 (88.1%) and 102 (89.5%) of the determined pesticides by UHPLC-MS/MS and GC-MS/MS, respectively, had RSDs in the interval 5–15% which demonstrates the good repeatability of both methods.

LOQ values, defined as the lowest concentration or mass of the analyte that has been validated with acceptable accuracy by applying the complete analytical method and identification criteria by SANTE Guidance [20], were found in the range 2.60–21.39 µg/kg for those analytes determined by LC-MS/MS and in the range 9.22–17.22 µg/kg for those analysed by GC-MS/MS. The LOQ values for all the pesticides analysed were lower than the MRLs established for these compounds in wine grapes, except for carbaryl which was 2 times its respective MRL.

### 3.2. Evaluation of Pesticide Residues Occurrence in Red Wine Samples

After method validation, the developed procedures were used to evaluate the occurrence of pesticides in 84 red wines from the Canary Islands, the Iberian Peninsula and Cape Verde. For this purpose, additional on-going validation was carried out by injecting four matrix-matched standards in the concentration range previously studied, and checking the recovery at one concentration level. In these cases, linearity must present an R^2^ higher than 0.9900, and recovery values in the range 60–140% with RSDs lower than 20%. Each pesticide was determined using the appropriate method and the obtained concentrations are provided as Supplementary Information (Table S4).

Most of analysed pesticides (142; 82.1% of all pesticides studied) were not detected in any of the red wine samples evaluated. This percentage was higher than the percentage (46%) found by Castro et al. [21] for 25 wines commercialised in Germany from several countries. However, only three red wines, 3.6% of the analysed samples, had not any of the evaluated compounds.

In the present study, only four samples (4.8%) presented one residue, while multiple residues were found in most of them (77; 91.7%). It should be highlighted that 49 red wines (58.3%) contained more than six pesticide residues, among which in 24 samples (28.6%) ten or more residues were detected, and in one wine up to 15 compounds.

**Figure 1:**
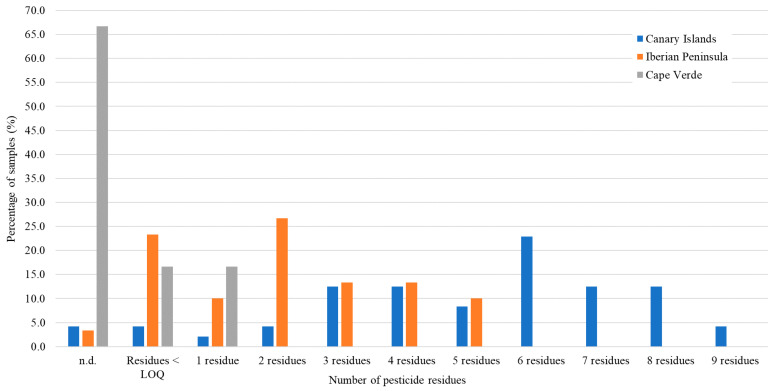
Histogram of the distribution of red wine samples according to the number of pesticide residues detected, differentiating between the Canary Islands (*n* = 48), the Iberian Peninsula (*n* = 30), and Cape Verde (*n* = 6) regions.

The number of pesticides detected in red wine samples according to their origin (the Canary Islands and the Iberian Peninsula) is shown in Figure 1. The red wines from Cape Verde were not included in this figure because of the low number of samples analysed. Clear differences were observed between both, the red wines from the Canary Islands and those from the Spanish Mainland. Eighteen (60% of the total) red wines from the Iberian Peninsula had not any or only had two or less pesticide residues, while the red wines from the Canary Islands with these numbers of pesticides represented only 11% of the total. In contrast, there were not red wine samples from the Iberian Peninsula with six or more residues quantified, while most of red wines from the Canary Islands (25; 52%) did present six or more different quantified residues. The wine samples from the Canary Islands had a higher number and variety of pesticides than the samples from the Iberian Peninsula. Likewise, the amounts of these compounds in the Canarian red wines were also higher since their occurrence could be quantified in a greater number of samples.

**Figure 2:**
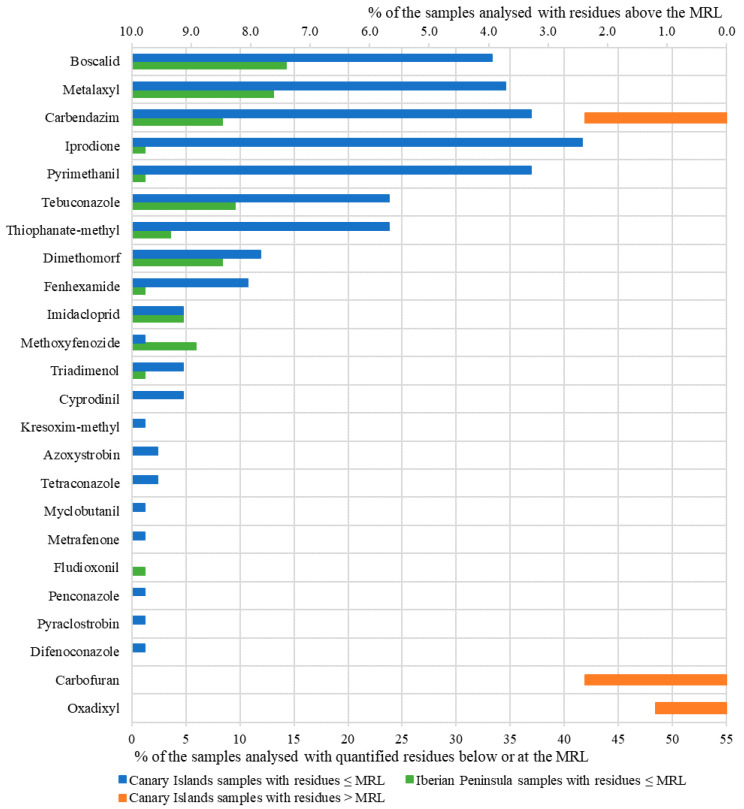
Percentage of the Canary Islands and the Iberian Peninsula red wine samples, over total samples, with quantified residues below or equal to their maximum residue limits (MRLs) and with residues above the MRL set for wine grapes (default processing factor (PF) of 1 applied according to Regulation (EU) No 2015/595).

From a quantitative point of view, Figure 2 shows the number and frequency considering three levels of concentrations (below LOQ; between LOQ and MRL; and above MRL) for the 31 pesticide residues that were detected in at least one red wine sample. Two origins of the red wines, the Canary Islands and the Iberian Peninsula were clearly differentiated in this figure. Sixty-eight red wine samples (81% of the total) contained one or several pesticides in quantified concentrations. Only two residues were detected in the red wine samples from Cape Verde, kresoxim-methyl and tebuconazole, which were found in all samples. Additionally, dimetomorph was only detected in a single sample. The concentration levels of these analytes were extremely low in all the analysed wines from Cape Verde.

Most of pesticides evaluated were found at concentrations below 10% of their corresponding MRL in vinification grapes, which agrees with other studies [21]. However, three of them exceeded the maximum established limit (MRL) in one or several red wine samples. All of them proceeded from the Canary Islands. Two red wines exceeded the MRL (0.5 mg/kg in EU) for the not approved pesticide carbendazim. This pesticide was detected in most of the analysed red wines (57; 67.9%), which is usual due to the use of this active pesticide in both Spanish regions. There were three wines in which carbofuran (2) and oxadixyl (1) were detected; the corresponding MRLs established in EU (0.002 and 0.01 mg/kg, respectively) were also exceeded in these three samples.

Tebuconazole, boscalid, carbendazim, metalaxyl, iprodione, dimethomorph, thiophanate-methyl and pyrimethanil were the most abundant pesticides, being detected in more than half of the red wine samples. Castro et al. [21] found these pesticide residues, except iprodione and thiophanate-methyl, within the 12 residues present in more than 24% of the commercial German wines analysed. This suggests the ubiquity of these residues in wines from these regions. Apart from that, a similar profile of detected residues was found for red wines produced in both Spanish regions. This fact could be associated with the use of similar groups of pesticides in both locations. However, remarkable differences from a quantitative point of view could be observed. In the case of the Canary Islands wines, positive samples ranged between 65%, found for thiophanate-methyl, and 83.3%, for iprodione and carbendazim. For the Spanish Mainland, these percentages decreased to 46.7% and 70.0% for metalaxyl and tebuconazole, respectively. Table 2 shows the mean concentrations of these eight pesticide residues for all the red wine samples and differentiates them according to their origin. The mean concentrations of these eight pesticides in red wines from the Canary Islands were higher than the mean concentrations obtained in red wines from the Iberian Peninsula, except for dimethomorph. A higher percentage of red wines with detectable concentrations were found in samples from the Canary Islands. Moreover, it can be observed that, except for methoxyfenozide and fludioxonil, a greater number of samples from the Canary Islands could be quantified, which agrees with results previously indicated. Remarkable differences were observed for iprodione and pyrimethanil between the red wines from the Canary Islands and the Iberian Peninsula.

Only one paper [13] has been published concerning the pesticide residue contents of red wine from the Canary Islands. Eleven pesticides were analysed in 18 home-made red wines from this region and two commercial red wines from the Iberian Peninsula. None of the pesticides analysed was detected in the Iberian Peninsula red wines. Procymidone was detected in 67% of the red wines from the Canary Islands, which contrasts with the results obtained in this work, in which this pesticide was not found in either the Canary Islands or the Iberian Peninsula samples. Pyrimethanil was detected in 39% of the red wines from the Canary Islands, while azoxystrobin was only detected in one red wine. In this work, both pesticides were the most frequently detected, 70.8% and 27.0%, respectively. This could be related to the lower LOQ achieved in this work using highly sensitive systems (UHPLC-MS/MS and GC-MS/MS) or due to a higher pesticide contamination of the current red wines.

Regarding the studies in which the occurrence of pesticides in red wines from other regions of Spain has been assessed, Castro et al. [14] detected pyrimethanil in 81.3% and cyprodinil in 75.0% of the total analysed Galician red wine samples. Pérez-Mayán et al. [15] quantified imidacloprid in 48% of the total evaluated Galician wines (both red and white wines), while Rodríguez-Cabo et al. [16] found fenhexamid in 18.2%, pyrimethanil in 27.3%, triadimenol and tebuconazole in 36.4%, metalaxyl, cyprodinil and azoxystrobin in 45.5%, and iprodione in 81.8% of the total analysed red wine samples from different Spanish geographic locations. In the same way, Pérez-Ortega et al. [17] found fenhexamid in 4.2%, penconazole in 8.3%, carbendazim in 16.7%, dimethomorph in 20.8%, tebuconazole in 25.0%, pyrimethanil in 29.2%, azoxystrobin in 33.3% and metalaxyl in 41.7% of 24 studied red wines from several regions of Peninsular Spain. In the case of the South, red wine samples were evaluated by Romero-González et al. [18]; none of the pesticides quantified in their work were in common with those detected in the present work. In this regard, it is noteworthy to mention that penconazole, azoxystrobin and cyprodinil were not quantified in the red wines from the Spanish Mainland analysed in this study, however, those pesticide residues were found in wines from the Canary Islands in a percentage of 1.2%, 2.4% and 4.8% of the total samples analysed, respectively. The rest of pesticides mentioned, triadimenol, fenhexamid, pyrimethanil and iprodione, were found in 1.2%, imidacloprid in 4.8%, dimethomorph and carbendazim in 8.3%, tebuconazole in 9.5% and metalaxyl in 13.1% of the total analysed red wine samples from the Spanish Peninsula, as well as being found in the range of 4.8–41.7% for the evaluated Canarian red wines.

Regarding the presence of pesticides in red wines produced in Cape Verde, no literature data was available. Therefore, the results informed in this work can serve as a reference for future studies.

### 3.3. Potential Toxicity of Pesticide Residues by the Red Wine Consumption

A comparison of the estimated dietary exposure to pesticides through red wine consumption with the long-term (chronic) risk toxicological reference values was developed in order to evaluate the health risk of the consumers. The tolerable daily intake (TDI) is the toxicological parameter used for long-term toxicity [22]. Two scenarios are usually considered: (1) The upper-bound scenario, which overestimates the real risk. Pesticide residues with concentrations below the LOQ have been assigned the LOQ value. (2) The lower-bound scenario, which underestimates the real risk. Those present at levels below the LOQ have been considered as not detected. Table 3 shows the long-term toxicity for the detected pesticides. A maximum tolerable amount of alcohol of 40 g/day for adults was considered; above this threshold, chronic toxicological effects are commonly produced in the consumers [23]. It is necessary to have a consumption of 360 g of wine (14°)/day (around 360 mL) to reach that amount of ethanol. Taking this data as a reference, and considering the upper-bound exposure assessment (first scenario), it can be deduced that the consumption of the red wine samples should contribute to the TDI in a percentage lower than 0.3%. In fact, only carbedazim, carbofuran, and iprodione presented percentages higher than 0.5%. Iprodione can be emphasised because its use was not approved by the European Commission (EC) from January 2019 [24], so the occurrence of such pesticide in the evaluated samples is particularly troubling. Despite the presence of a large number of pesticides in red wines produced in the Canary Islands and Peninsular Spain and having a large number of samples that contain 10 or more pesticide residues and even certain residues that reach values of 28% and 24% of the TDI, as in the case of carbenzamin and carbofuran, respectively, it can be stated that, individually (for each residue, without considering a possible cumulative effect), the consumption of these wines does not entail a risk to consumer health. In this way, it can be concluded that the probability of being exposed to pesticides exceeding concentrations that may lead to negative health effects is low.

## 4. Conclusions

The QuEChERS method coupled with UHPLC-(Q-ToF)-MS/MS and GC-QqQ-MS/MS were tested and optimised for the evaluation of pesticide occurrence in red wine samples. The proposed methodologies provided adequate accuracy and precision, as well as good sensitivity, with low LOQs, in the range 2.60–21.39 µg/kg, for the analysis of a large number of substances (*n* = 173) commonly used in the growing of grapes, which guarantee the reliability of the obtained results.

A considerable number of pesticides were detected and quantified in red wines from different origins (the Canary Islands, the Iberian Peninsula and Cape Verde). Samples from the Canary Islands and the Iberian Peninsula showed a similar profile of contamination from a qualitative point of view. However, Canarian wines presented the biggest variety of residues and the highest occurrence, which is in accordance with the larger use of phytosanitary products in this region in recent decades. In contrast, the number, and concentrations of pesticide residues in red wines from Cape Verde were very low, indicating a lower contamination incidence in products coming from this region.

Regarding the potential risk of pesticide exposure to human health, associated with a high or regular wine consumption, the obtained results indicate that, although some of analysed samples contribute with relative high percentages for certain pesticides (carbedazim, carbofuran) the exposure is still low. However, the occurrence of a great variety of residues, particularly in the analysed Spanish wines, makes it necessary to maintain a continuous monitoring of these products to ensure consumer safety.

## Figures and Tables

**Table 1:** Validation data of detected pesticides by (i) UHPLC-(Q-ToF)-MS/MS and (ii) GC-QqQ-MS/MS in red wine.

**(i) Pesticide**	**Calibration Data (*n* = 7)**		**Relative Recovery ^a^ (*n* = 5) (RSD, %)**	**LOQ_method_^b^ (µg/kg)**
	**Range of Concentration Studied (µg/L)**	**R^2^**		
Azoxystrobin	10–150	0.9950	94 (13)	11.76
Carbendazim ^d^	10–150	0.9970	89 (14)	12.13
Carbofuran ^d^	2–150	0.9996	89 (14)	2.60
Diethofencarb	10–150	0.9982	84 (13)	13.18
Dimethomorph I ^e^	10–150	0.9951	76 (20)	15.42
Dimethomorph II ^e^	10–150	0.9946	85 (11)	12.76
Fenhexamid	10–150	0.9989	88 (12)	12.41
Imidacloprid	10–150	0.9961	80 (15)	14.82
Iprovalicarb	15–150	0.9957	94 (12)	17.66
Methoxyfenozide	10–150	0.9982	91 (14)	12.47
Pyraclostrobin	10–150	0.9936	85 (14)	13.35
Thiophanate-methyl	10–150	0.9982	75 (12)	15.57
**(ii) Pesticide**	**Calibration Data (*n* = 7)**		**Relative Recovery ^c^ (*n* = 10) (RSD, %)**	**LOQ_method_^b^ (µg/kg)**
	**Range of Concentration Studied (µg/L)**	**R^2^**		
Benalaxyl	10–200	0.9940	79 (10)	12.85
Boscalid	10–200	0.9970	85 (15)	12.10
Chlorpyrifos-methyl	10–200	0.9921	79 (13)	13.19
Cyprodinil	10–200	0.9991	77 (12)	13.53
Difenoconazole	10–200	0.9946	83 (15)	12.75
Fenbuconazole	10–200	0.9919	80 (12)	12.89
Fludioxonil	10–200	0.9949	77 (13)	13.17
Iprodione ^d^	10–200	0.9983	91 (11)	11.18
Kresoxim-methyl	10–200	0.9975	94 (13)	11.01
Metalaxyl	10–200	0.9994	115 (19)	9.35
Metrafenone	10–200	0.9937	99 (8)	10.31
Myclobutanil	10–200	0.9969	96 (17)	10.73
Nuarimol ^d^	10–200	0.9972	89 (14)	11.69
Oxadixyl ^d^	10–200	0.9968	83 (14)	12.43
Penconazole ^f^	10–200	0.9965	84 (14)	12.52
Pyrimethanil	10–200	0.9980	111 (17)	9.22
Tebuconazole	10–200	0.9989	95 (15)	11.37
Tetraconazole	10–200	0.9990	83 (15)	11.69
Triadimefon ^d^	10–200	0.9913	86 (14)	12.01
Triadimenol ^d,g^	10–200	0.9964	87 (13)	11.90

LOQ_method_: limit of quantification of the method; R^2^: Determination coefficient. Triphenylphosphate (TPP) was used as surrogate in all cases. ^a^ Concentration of target analytes was 75 µg/kg; ^b^ Defined as the lowest concentration of the analyte that has been validated with acceptable accuracy by applying the complete analytical method and identification criteria by SANTE Guidance (SANTE/12682/2019, 2019); ^c^ The average of 5 extractions at two different concentration levels (*n* = 10): 10 µg/kg and 100 µg/kg; ^d^ Pesticide not approved by the European Union (EU); ^e^ The sum of isomers expressed as dimethomorph; ^f^ The sum of constituent isomers; ^g^ Determined as any ratio of constituent isomers.

**Table 2:** Mean concentrations of the more abundant pesticides in the overall analysed red wine samples and their differentiation according to their origin.

Pesticide	X ± SD ^a^			*p* ^b^
	Total (*n* = 78)	Canary Islands (*n* = 48)	Iberian Peninsula (*n* = 30)	
Boscalid	0.010 ± 0.014 (51.3%)	0.014 ± 0.017 (58.3%)	0.005 ± 0.007 (40.0%)	0.022
Carbendazim	0.055 ± 0.139 (51.3%)	0.076 ± 0.165 (68.8%)	0.021 ± 0.074 (23.3%)	0.000
Dimethomorph	0.003 ± 0.008 (21.8%)	0.003 ± 0.009 (20.8%)	0.003 ± 0.008 (23.3%)	0.853
Iprodione	0.038 ± 0.076 (46.2%)	0.061 ± 0.089 (72.9%)	0.001 ± 0.005 (3.3%)	0.000
Metalaxyl	0.033 ± 0.066 (51.3%)	0.047 ± 0.080 (60.4%)	0.010 ± 0.015 (36.7%)	0.013
Pyrimethanil	0.028 ± 0.084 (41.0%)	0.044 ± 0.104 (64.6%)	0.001 ± 0.007 (3.3%)	0.000
Tebuconazole	0.010 ± 0.032 (35.9%)	0.014 ± 0.040 (41.7%)	0.004 ± 0.009 (26.7%)	0.131
Thiophanate-methyl	0.038 ± 0.159 (29.5%)	0.056 ± 0.200 (41.7%)	0.009 ± 0.031 (10.0%)	0.006

^a^ The average concentration (X) and standard deviation (SD) of each compound were calculated considering the pesticide residues below the limit of quantification (LOQ) of the method as non-detected. The percentage of quantified red wine samples is indicated between brackets; ^b^ The *p*-value was obtained for comparison between the Canary Islands and the Iberian Peninsula mean values by applying the Mann-Whitney U test.

**Table 3:** Long-term toxicity study of detected pesticides in the analysed red wine samples (*n* = 84).

Pesticide	TDI (mg/kg·bw·Day) ^b^	Canary Islands		Iberian Peninsula	
		Chronic Toxicity (%) ^c^			
		Lower Approach ^d^	Upper Approach ^e^	Lower Approach ^d^	Upper Approach ^e^
Azoxystrobin	0.2	0.011	0.018	-	0.009
Benalaxyl	0.04	-	0.003	-	0.005
Boscalid	0.04	0.203	0.238	0.080	0.120
Carbendazim ^a^	0.02	2.269	2.313	0.640	0.740
Carbofuran ^a^	0.00015	0.917	0.917	-	-
Chlorpyrifos-methyl ^a^	0.01	-	0.013	-	-
Cyprodinil	0.03	0.242	0.263	-	0.040
Diethofencarb	0.43	-	-	-	0.0005
Difenoconazole	0.01	0.013	0.013	-	-
Dimethomorph	0.05	0.040	0.075	0.040	0.080
Fenbuconazole	0.006	-	-	-	0.033
Fenhexamid	0.2	0.032	0.034	0.002	0.002
Fludioxonil	0.37	-	0.0003	0.0011	0.0043
Imidacloprid	0.06	0.017	0.025	0.030	0.047
Iprodione ^a^	0.02	1.819	1.838	0.030	0.060
Iprovalicarb	0.015	-	0.017	-	0.027
Kresoxim-methyl	0.4	0.0003	0.003	-	0.001
Metalaxyl	0.08	0.352	0.363	0.075	0.083
Methoxyfenozide	0.1	0.001	0.001	0.036	0.036
Metrafenone	0.25	0.0005	0.005	-	0.001
Myclobutanil	0.025	0.010	0.075	-	0.064
Nuarimol ^a^	-	-	-	-	-
Oxadixyl ^a^	-	-	-	-	-
Penconazole	0.03	0.004	0.017	-	-
Pyraclostrobin	0.03	0.008	0.008	-	-
Pyrimethanil	0.17	0.156	0.158	0.005	0.009
Tebuconazole	0.03	0.275	0.346	0.087	0.173
Tetraconazole	0.004	0.125	0.438	-	0.100
Thiophanate-methyl	0.08	0.420	0.438	0.068	0.085
Triadimefon ^a^	0.03	-	-	-	0.007
Triadimenol ^a^	0.05	0.030	0.035	0.008	0.008

bw: body weight; n.a.: not applicable; TDI: tolerable daily intake. ^a^ Pesticide not approved by the European Comission (EC); ^b^ Toxicological data obtained from the European Union (EU) Pesticides database (EU Pesticides database); ^c^ Defined as the percentage of the ratio between the average concentration of each pesticide detected in the red wine samples and the respective value of the TDI, considering an average body weight of 60 kg and a mass of wine of 360 g/day (considering a maximum tolerable amount of alcohol of 40 g/day). The equivalence factor of volume/mass of wine was considered as 1; ^d^ whereas those concentration values are below their respective LOQs are assigned as not detected (n.d.); ^e^ whereas those concentration values that are below their respective limits of quantification (LOQs) are assigned the LOQ value.

## Supplementary Materials

The following are available online at https://www.mdpi.com/2304-8158/9/11/1555/s1, Table S1: Summary of UHPLC-(Q-ToF)-MS/MS validation data of the studied compounds in red wine, Table S2: Summary of GC-QqQ-MS/MS validation data of the studied compounds in red wine, Table S3: Data of the red wine samples analysed (*n* = 84), Table S4: Concentrations obtained of the red wine samples analysed (*n* = 84). Figure S1: UHPLC-MS/MS base peak chromatogram of a standard solution spiked with the pesticides and TPP at a concentration level of 100 μg/L, Figure S2: GC-MS/MS total ion chromatogram of a standard solution spiked with the pesticides and TPP at a concentration level of 100 μg/L.
