# Supplementary material for: Comparison of Pesticide Residue Levels in Red Wines from Canary Islands, Iberian Peninsula, and Cape Verde
Source: Foods. 2020 Oct 27;9(11):1555. doi: 10.3390/foods9111555 (PMC7694142; doi:10.3390/foods9111555)
Supplement: Supplementary file 1 [file foods-09-01555-s001.pdf]

**Table S1.-** Summary of UHPLC-(Q-ToF)-MS/MS validation data of the studied compounds in red wine.

| Pesticide | Retention time (min) | Calibration data (n = 7) $R^2$ | Relative recovery[a)] (n = 5) (RSD, %) | $LOQ_{method}$[b)] (µg/kg) |
|---|---|---|---|---|
| | | Acaricide | | |
| Fenpyroximate | 11.14 | 0.9977 | 63 (15) | 16.44 |
| | | Fungicide | | |
| Azoxystrobin | 8.19 | 0.9950 | 94 (13) | 11.76 |
| Bromuconazole I[d)] | 8.70 | 0.9975 | 71(16) | 16.01 |
| Bromuconalole II[d)] | 9.33 | 0.9986 | 90 (14) | 13.42 |
| Carbendazim[c)] | 4.71 | 0.9970 | 89 (14) | 12.13 |
| Cyazofamid | 9.05 | 0.9993 | 88 (13) | 12.60 |
| Diethofencarb | 8.08 | 0.9982 | 84 (13) | 13.18 |
| Dimethomorph I[e)] | 8.36 | 0.9951 | 76 (20) | 15.42 |
| Dimethomorph II[e)] | 8.64 | 0.9946 | 85 (11) | 12.76 |
| Fenhexamid | 8.87 | 0.9989 | 88 (12) | 12.41 |
| Flusilazole[c)] | 9.26 | 0.9946 | 96 (10) | 15.56 |
| Flutriafol | 7.34 | 0.9991 | 82 (15) | 20.02 |
| Hexaconazole[c)] | 9.75 | 0.9983 | 91 (13) | 11.98 |
| Imazalil | 9.52 | 0.9970 | 108 (8) | 10.14 |
| Iprovalicarb | 8.84 | 0.9957 | 94 (12) | 17.66 |
| Mandipropamid | 8.49 | 0.9931 | 89 (16) | 12.20 |
| Mepanipyrim | 8.75 | 0.9999 | 96 (9) | 17.20 |
| Pencycuron | 9.94 | 0.9949 | 78 (12) | 13.59 |
| Prochloraz | 9.83 | 0.9992 | 94 (13) | 12.99 |
| Spiroxamine[f)] | 10.50 | 0.9988 | 75 (14) | 15.89 |
| Thiabendazole | 5.42 | 0.9982 | 85 (14) | 12.93 |
| Thiophanate-methyl | 6.22 | 0.9982 | 75 (12) | 15.57 |
| Trifloxystrobin | 10.17 | 0.9982 | 78 (12) | 13.74 |
| Triticonazole | 8.98 | 0.9991 | 87 (10) | 12.64 |
| | | Herbicide | | |
| Bensulfuron | 6.72 | 0.9985 | 73 (15) | 16.62 |
| Chlorotoluron | 6.97 | 0.9994 | 80 (13) | 13.72 |
| Flazasulfuron | 4.58 | 0.9990 | 67 (12) | 17.14 |
| Florasulam | 4.21 | 0.9992 | 70 (12) | 17.12 |
| Isoproturon[c)] | 7.37 | 0.9989 | 72 (16) | 15.63 |
| Isoxaben | 8.54 | 0.9997 | 89 (12) | 13.25 |
| Linuron[c)] | 8.01 | 0.9994 | 92 (14) | 11.64 |
| Mesosulfuron[g)] | 4.89 | 0.9986 | 78 (15) | 14.77 |
| Phenmedipham | 7.82 | 0.9977 | 87 (16) | 12.74 |
| Pinoxaden | 9.88 | 0.9993 | 78 (8) | 15.04 |
| Triasulfuron[c)] | 4.83 | 0.9986 | 61 (9) | 19.69 |
| Tribenuron[h)] | 4.77 | 0.9970 | 100 (12) | 11.12 |
| | | Insecticide | | |
| Acetamiprid | 4.42 | 0.9980 | 73 (12) | 16.18 |
| Chlorantraniliprole | 7.84 | 0.9997 | 84 (12) | 13.31 |
| Diflubenzuron | 9.14 | 0.9994 | 86 (16) | 12.95 |
| Flubendiamide[d)] | 9.43 | 0.9985 | 91 (12) | 12.06 |

| | | | | |
|---|---|---|---|---|
| Flufenoxuron[c)] | 11.04 | 0.9996 | 71 (16) | 15.32 |
| Hexaflumuron[c)] | 10.18 | 0.9989 | 83 (14) | 14.16 |
| Imidacloprid | 3.93 | 0.9961 | 80 (15) | 14.82 |
| Lufenuron | 10.70 | 0.9943 | 95 (11) | 11.61 |
| Methoxyfenozide | 8.58 | 0.9982 | 91 (14) | 12.47 |
| Pymetrozine[c)] | 3.21 | 0.9964 | 87 (13) | 12.32 |
| Tebufenozide | 9.30 | 0.9901 | 80 (14) | 13.87 |
| Teflubenzuron[d)] | 10.60 | 0.9988 | 88 (13) | 12.62 |
| Thiacloprid | 4.93 | 0.9970 | 99 (13) | 10.60 |
| Thiamethoxam[c)] | 3.20 | 0.9991 | 102 (12) | 21.39 |
| Thiodicarb[c)] | 6.95 | 0.9996 | 91 (9) | 12.80 |
| | | Nematicide | | |
| Fosthiazate | 7.00 | 0.9997 | 86 (11) | 13.29 |
| | | Plant Growth Regulator | | |
| Paclobutrazol | 8.47 | 0.9988 | 89 (12) | 12.64 |
| | | Acaricide/Insecticide | | |
| Hexythiazox | 10.81 | 0.9986 | 89 (14) | 13.52 |
| Spirodiclofen | 11.17 | 0.9997 | 85 (7) | 12.63 |
| | | Fungicide/Plant Growth Regulator | | |
| Metconazole | 9.77 | 0.9995 | 86 (12) | 13.16 |
| Pyraclostrobin | 9.72 | 0.9936 | 85 (14) | 13.35 |
| | | Insecticide/Plant Growth Regulator | | |
| Carbaryl[c)] | 6.61 | 0.9984 | 83 (11) | 20.67 |
| | | Acaricide/Insecticide/Nematicide | | |
| Carbofuran[c)] | 6.35 | 0.9996 | 89 (14) | 2.60 |

$LOQ_{method}$: limit of the quantification of the method; $R^2$: Determination coefficient. TPP was used as surrogate in all cases. Range of concentration studied was 10-150 µg/L, except for carbofuran which was 2-150 µg/L; carbaryl, flutriafol, iprovalicarb and mepanipyrim which was 15-150 µg/L; and thiamethoxam which was 20-150 µg/L. a) Concentration of target analytes was 75 µg/kg. b) Defined as the lowest concentration or mass of the analyte that has been validated with acceptable accuracy by applying the complete analytical method and identification criteria by SANTE Guidance (SANTE/12682/2019, 2019). c) Pesticide not approved by the European Union (EU). d) Sum of diasteroisomers expressed as bromuconazole. e) Sum of isomers expressed as dimethomorph. f) Sum of isomers. g) Mesosulfuron-methyl expressed as mesosulfuron. h) Tribenuron-methyl expressed as tribenuron.

**Table S2.-** Summary of GC-QqQ-MS/MS validation data of the studied compounds in red wine.

| Pesticide | Retention time (min) | Calibration data (n = 7) $R^2$ | Relative recovery[a] (n = 10) (RSD, %) | $LOQ_{method}$[b] (µg/kg) |
|---|---|---|---|---|
| 4,4'-DCBP[d] | 9.88 | 0.9963 | 85 (8) | 12.03 |
| | | Acaricide | | |
| Acrinathrin | 14.90 | 0.9915 | 105 (16) | 11.31 |
| Bromopropylate[c] | 13.76 | 0.9938 | 90 (11) | 11.33 |
| Fenazaquin | 14.05 | 0.9961 | 88 (12) | 11.78 |
| Tebufenpyrad | 13.95 | 0.9978 | 97 (6) | 10.48 |
| | | Fungicide | | |
| Benalaxyl | 12.71 | 0.9940 | 79 (10) | 12.85 |
| Boscalid | 16.43 | 0.9970 | 85 (15) | 12.10 |
| Bupirimate | 11.66 | 0.9968 | 84 (13) | 12.34 |
| Chlorothalonil[c] | 8.45 | 0.9946 | 60 (5) | 16.99 |
| Cyproconazole | 11.86 | 0.9944 | 73 (15) | 14.14 |
| Cyprodinil | 10.25 | 0.9991 | 77 (12) | 13.53 |
| Dichlofluanid[c] | 9.61 | 0.9963 | 72 (12) | 14.38 |
| Dicloran[c] | 7.68 | 0.9978 | 77 (13) | 13.46 |
| Difenoconazole | 17.73 | 0.9946 | 83 (15) | 12.75 |
| Diniconazole[c] | 12.15 | 0.9946 | 74 (15) | 13.30 |
| Epoxiconazole | 13.41 | 0.9952 | 84 (13) | 12.28 |
| Fenamidone[c] | 13.99 | 0.9973 | 85 (16) | 11.94 |
| Fenarimol[c] | 14.92 | 0.9949 | 80 (11) | 12.75 |
| Fenbuconazole | 16.06 | 0.9919 | 80 (12) | 12.89 |
| Fludioxonil | 11.39 | 0.9949 | 77 (13) | 13.17 |
| Kresoxim-methyl | 11.67 | 0.9975 | 94 (13) | 11.01 |
| Metalaxyl | 9.20 | 0.9994 | 115 (19) | 9.35 |
| Metrafenone | 15.18 | 0.9937 | 99 (8) | 10.31 |
| Myclobutanil | 11.60 | 0.9969 | 96 (17) | 10.73 |
| Nuarimol[c] | 13.13 | 0.9972 | 89 (14) | 11.69 |
| Ofurace[c] | 12.62 | 0.9953 | 87 (15) | 11.93 |
| Oxadixyl[c] | 12.27 | 0.9968 | 83 (14) | 12.43 |
| Penconazole[e] | 10.40 | 0.9965 | 84 (14) | 12.52 |
| Procymidone[e] | 10.69 | 0.9930 | 95 (13) | 10.99 |
| Propiconazole[c], [f] | 12.78 | 0.9945 | 82 (11) | 12.69 |
| Pyrazophos[c] | 14.98 | 0.9954 | 104 (15) | 9.85 |
| Pyrimethanil | 8.17 | 0.9980 | 111 (17) | 9.22 |
| Quinoxyfen[c] | 12.76 | 0.9924 | 85 (6) | 12.02 |
| Tebuconazole | 13.11 | 0.9989 | 95 (15) | 11.37 |
| Tetraconazole | 9.92 | 0.9990 | 83 (15) | 11.69 |
| Tolclofos-methyl | 9.09 | 0.9967 | 89 (10) | 11.65 |
| Triadimefon[c] | 9.86 | 0.9913 | 86 (14) | 12.01 |
| Triadimenol[c], [g] | 10.70 | 0.9964 | 87 (13) | 11.90 |
| Triflumizole[c] | 10.71 | 0.9949 | 87 (12) | 11.80 |
| Vinclozolin[c] | 8.98 | 0.9956 | 88 (10) | 11.75 |
| | | Herbicide | | |
| Clomazone | 7.87 | 0.9944 | 77 (12) | 13.49 |
| Diflufenican | 13.16 | 0.9951 | 97 (13) | 10.84 |

| | | | | |
|---|---|---|---|---|
| Dimethenamid[c)] | 8.89 | 0.9966 | 77 (13) | 13.49 |
| Ethofumesate | 9.48 | 0.9938 | 88 (14) | 12.12 |
| Metribuzin | 8.86 | 0.9957 | 85 (15) | 12.09 |
| Oxadiazon[c)] | 11.50 | 0.9974 | 94 (12) | 11.03 |
| Oxyfluorfen | 11.57 | 0.9922 | 95 (13) | 10.86 |
| Pendimethalin | 10.37 | 0.9954 | 93 (13) | 11.17 |
| Prometryn[c)] | 9.15 | 0.9957 | 87 (14) | 11.95 |
| Propanil[c)] | 8.81 | 0.9971 | 86 (14) | 12.01 |
| Propyzamide | 8.06 | 0.9929 | 86 (15) | 12.01 |
| Simazine[c)] | 7.70 | 0.9966 | 82 (12) | 12.57 |
| Terbutryn[c)] | 9.38 | 0.9959 | 86 (12) | 12.00 |
| Trifluralin[c)] | 7.16 | 0.9940 | 86 (11) | 11.80 |
| Insecticide | | | | |
| Bendiocarb[c)] | 7.17 | 0.9958 | 82 (16) | 12.82 |
| Chlorfenvinphos[c)] | 10.52 | 0.9961 | 79 (14) | 13.08 |
| Deltamethrin | 17.98 | 0.9901 | 92 (16) | 11.97 |
| Etofenprox | 16.64 | 0.9935 | 93 (11) | 11.23 |
| Fenthion[c)] | 9.78 | 0.9940 | 90 (12) | 11.47 |
| Fipronil[c)] | 10.51 | 0.9952 | 87 (15) | 12.13 |
| Fonofos[c)] | 8.13 | 0.9969 | 78 (11) | 13.35 |
| Indoxacarb | 17.90 | 0.9939 | 91 (16) | 11.75 |
| Isocarbophos[c)] | 9.93 | 0.9962 | 82 (13) | 12.73 |
| Isofenphos-methyl[c)] | 10.26 | 0.9940 | 83 (15) | 12.44 |
| lambda-Cyhalothrin[h)] | 14.74 | 0.9912 | 97 (13) | 10.35 |
| Permethrin[c), f)] | 15.46 | 0.9960 | 98 (9) | 10.57 |
| Phenthoate[c)] | 10.59 | 0.9945 | 94 (14) | 10.83 |
| Pirimicarb | 8.60 | 0.9969 | 85 (14) | 12.11 |
| Pirimiphos-ethyl[c)] | 10.16 | 0.9924 | 100 (14) | 10.10 |
| Pirimiphos-methyl | 9.45 | 0.9946 | 96 (11) | 10.84 |
| Profenofos[c)] | 11.39 | 0.9936 | 95 (8) | 10.70 |
| Propoxur[c)] | 6.75 | 0.9921 | 87 (11) | 11.89 |
| Pyriproxyfen | 14.47 | 0.9968 | 112 (11) | 9.61 |
| Quinalphos[c)] | 10.58 | 0.9934 | 91 (10) | 11.18 |
| tau-Fluvalinate | 17.45 | 0.9925 | 89 (16) | 11.97 |
| Tefluthrin | 8.31 | 0.9961 | 86 (10) | 11.99 |
| Tetrachlorvinphos[c)] | 11.00 | 0.9951 | 97 (10) | 10.48 |
| Nematicide | | | | |
| Fenamiphos | 11.17 | 0.9927 | 97 (15) | 10.31 |
| Acaricide/Insecticide | | | | |
| Bifenthrin[c), f)] | 13.77 | 0.9949 | 89 (12) | 11.68 |
| Buprofezin | 11.62 | 0.9966 | 80 (11) | 13.21 |
| Chlorfenapyr[c)] | 11.90 | 0.9954 | 92 (14) | 11.20 |
| Chlorpyrifos | 9.81 | 0.9936 | 81 (9) | 12.78 |
| Chlorpyrifos-methyl | 9.01 | 0.9921 | 79 (13) | 13.19 |
| Cyfluthrin[c), i)] | 16.12 | 0.9960 | 94 (13) | 11.01 |
| Cypermethrin[j)] | 16.44 | 0.9925 | 97 (16) | 10.99 |
| Diazinon[c)] | 8.17 | 0.9930 | 79 (12) | 13.11 |
| Dichlorvos[c)] | 4.66 | 0.9964 | 70 (10) | 15.12 |
| Dimethoate[d)] | 7.67 | 0.9967 | 71 (13) | 14.85 |

| | | | | |
|---|---|---|---|---|
| Endosulfan-alpha[c), k)] | 11.10 | 0.9950 | 72 (6) | 14.44 |
| Endosulfan-beta[c), k)] | 12.10 | 0.9962 | 78 (12) | 13.35 |
| Endosulfan-sulphate[c), k)] | 12.85 | 0.9949 | 59 (12) | 17.22 |
| Ethion[c)] | 12.27 | 0.9958 | 86 (16) | 12.00 |
| Fenitrothion[c)] | 9.45 | 0.9931 | 86 (12) | 11.95 |
| Fenpropathrin[c)] | 13.90 | 0.9956 | 89 (10) | 11.61 |
| Fenvalerate[c), l)] | 17.46 | 0.9915 | 92 (14) | 11.18 |
| Malathion | 9.60 | 0.9976 | 98 (11) | 10.45 |
| Methidathion[c)] | 10.84 | 0.9990 | 104 (13) | 9.99 |
| Mevinphos[c)] | 5.56 | 0.9964 | 76 (12) | 13.38 |
| Parathion[c), m)] | 9.83 | 0.9946 | 95 (11) | 10.69 |
| Phosalone[c)] | 14.42 | 0.9978 | 110 (11) | 9.44 |
| Pyridaben | 15.62 | 0.9946 | 96 (9) | 10.59 |
| Pyridaphenthion[c)] | 13.65 | 0.9929 | 110 (9) | 9.54 |
| Spiromesifen | 13.56 | 0.9955 | 98 (12) | 10.33 |
| Tetradifon[c)] | 14.25 | 0.9933 | 90 (9) | 11.46 |
| Triazophos[c)] | 12.49 | 0.9940 | 98 (15) | 10.83 |
| Acaricide/Fungicide | | | | |
| Quinomethionate[c)] | 10.86 | 0.9936 | 67 (16) | 15.57 |
| Tolylfluanid[c)] | 10.48 | 0.9948 | 83 (12) | 12.30 |
| Fungicide/Nematicide | | | | |
| Iprodione[c)] | 13.55 | 0.9983 | 91 (11) | 11.18 |
| Herbicide/Plant Growth Regulator | | | | |
| Chlorpropham | 7.02 | 0.9922 | 77 (12) | 13.12 |
| Propham[c)] | 5.72 | 0.9971 | 84 (13) | 12.65 |
| Insecticide/Nematicide | | | | |
| Cadusafos[c)] | 7.32 | 0.9964 | 77 (12) | 13.47 |
| Ethoprophos[c)] | 6.93 | 0.9929 | 76 (12) | 13.53 |
| Insecticide/Repellant | | | | |
| Parathion-methyl[c)] | 9.00 | 0.9984 | 93 (12) | 10.69 |
| Insecticide/Rodenticide | | | | |
| Lindane[c), n)] | 8.02 | 0.9963 | 88 (11) | 11.88 |

$LOQ_{method}$: limit of the quantification of the method; $R^2$: Determination coefficient. TPP was used as surrogate in all cases. Range of concentration studied was 10-200 µg/L. a) Average of 5 extractions at two different concentration levels (n =10): 10 µg/kg and 100 µg/kg. b) Defined as the lowest concentration or mass of the analyte that has been validated with acceptable accuracy by applying the complete analytical method and identification criteria by SANTE (SANTE/12682/2019, 2019). c) Pesticide not approved by the European Union (EU). d) 4,4´-Dichlorobenzophenone (4,4´-DCBP) is not part of the maximum residue limit (MRL) residue definition of dicofol, can still serve as a valuable indicator of dicofol presence. e) Sum of constituent isomers. f) Sum of isomers. g) Determined as any ratio of constituent isomers. h) Sum of R,S and S,R isomers. i) Cyfluthrin including other mixtures of constituent isomers (sum of isomers). j) Cypermethrin including other mixtures of constituent isomers (sum of isomers). k) Sum of alpha- and beta-isomers and endosulfan-sulphate expresses as endosulfan. l) Any ratio of constituent isomers (RR, SS, RS and SR) including esfenvalerate. m) Parathion-ethyl expressed as parathion. n) Gamma-isomer of hexachlorocyclohexane.

**Table S3.-** Data of the red wine samples analysed (n = 84).

| Sample Code | Type of red wine | Origin | Harvest year |
|---|---|---|---|
| CI 1 | - | San Miguel de Abona, Tenerife | 2013 |
| CI 2 | - | La Orotava, Tenerife | 2016 |
| CI 3 | Listán Negro and Negramoll | Tegueste, Tenerife | 2015 |
| CI 4 | - | Tijarafe, La Palma | 2016 |
| CI 5 | Listán Negro | Lanzarote | 2015 |
| CI 6 | Listán Negro and Tintilla | Gran Canaria | 2016 |
| CI 7 | - | Tacoronte, Tenerife | 2016 |
| CI 8 | Listán Negro 90%, vintage | Tacoronte, Tenerife | 2012 |
| CI 9 | - | Tacoronte, Tenerife | 2016 |
| CI 10 | - | Tacoronte, Tenerife | 2015 |
| CI 11 | Listán Negro, Negramoll, and Listán Blanco | Tegueste, Tenerife | 2015 |
| CI 12 | Listán Negro | La Orotava, Tenerife | 2016 |
| CI 13 | Listán Negro | La Orotava, Tenerife | 2015 |
| CI 14 | - | La Orotava, Tenerife | 2016 |
| CI 15 | Vijariego Negro | San Miguel de Abona, Tenerife | 2012 |
| CI 16 | Listán Negro and Negramoll | La Matanza de Acentejo, Tenerife | 2015 |
| CI 17 | Listán Negro | Las Palmas de Gran Canaria, Gran Canaria | 2015 |
| CI 18 | Listán negro, Merlot, and Syrah | Tacoronte, Tenerife | 2016 |
| CI 19 | - | Tacoronte, Tenerife | 2016 |
| CI 20 | - | Puntagorda, La Palma | 2015 |
| CI 21 | Listán Negro and Vijariego Negro | La Orotava, Tenerife | 2016 |
| CI 22 | - | Arafo, Tenerife | 2015 |
| CI 23 | - | Arico, Tenerife | 2016 |
| CI 24 | - | Tacoronte, Tenerife | 2015 |
| CI 25 | Listán Negro | La Orotava, Tenerife | 2001 |
| CI 26 | - | Tacoronte, Tenerife | 2017 |
| CI 27 | Listán Negro | Tenerife | 2017 |
| CI 28 | - | Tenerife | 2017 |
| CI 29 | - | Tenerife | 2017 |
| CI 30 | - | Tenerife | 2017 |
| CI 31 | - | Tenerife | 2015 |
| CI 32 | - | Las Palmas de Gran Canaria, Gran Canaria | 2017 |
| CI 33 | Listán Negro and Negramoll | Tenerife | 2017 |
| CI 34 | Listán Negro and Negramoll | Tenerife | 2012 |
| CI 35 | Listán Negro and Syrah | Tenerife | 2016 |
| CI 36 | - | Gran Canaria | 2015 |
| CI 37 | - | Lanzarote | - |
| CI 38 | - | Tenerife | 2016 |
| CI 39 | - | Tenerife | 2017 |

| | | | |
|---|---|---|---|
| CI 40 | - | Tenerife | 2016 |
| CI 41 | Listán Negro | Tenerife | 2017 |
| CI 42 | Negramoll | Tenerife | 2017 |
| CI 43 | - | Tenerife | 2016 |
| CI 44 | Listán Negro | Tenerife | 2017 |
| CI 45 | - | Tenerife | 2017 |
| CI 46 | - | Tenerife | 2017 |
| CI 47 | - | Tenerife | 2017 |
| CI 48 | Negramoll, Castellana Negra, Vijariego Negro, and Almuñeco | La Palma | 2017 |
| IP 1 | Tempranillo | Burgos, Castile and León | 2016 |
| IP 2 | - | Sant Sadurní D'Anoia, Catalonia | 2015 |
| IP 3 | Tempranillo | Burgos, Castile and León | 2016 |
| IP 4 | Tempranillo | Haro, La Rioja | 2016 |
| IP 5 | Vintage | Haro, La Rioja | 2014 |
| IP 6 | Tempranillo | Valladolid, Castile and León | 2013 |
| IP 7 | Tempranillo | Zamora, Castile and León | 2015 |
| IP 8 | - | Alava, Autonomous Community of the Basque Country | 2016 |
| IP 9 | - | Burgos, Castile and León | 2014 |
| IP 10 | Tempranillo and Mazuelo | Fuenmayor, La Rioja | 2014 |
| IP 11 | Tempranillo | Burgos, Castile and León | 2016 |
| IP 12 | - | Badajoz, Extremadura | 2015 |
| IP 13 | Tempranillo | Valdepeñas, Castile-La Mancha | 2016 |
| IP 14 | Vintage | Aldeanueva de Ebro, La Rioja | 2014 |
| IP 15 | Tempranillo | Burgos, Castile and León | 2016 |
| IP 16 | Tempranillo | La Rioja | 2014 |
| IP 17 | Tempranillo | Castile-La Mancha | - |
| IP 18 | Tempranillo | Castile and León | 2017 |
| IP 19 | Tempranillo | Castile and León | - |
| IP 20 | Tempranillo | Valladolid, Castile and León | 2016 |
| IP 21 | Tempranillo | La Rioja | 2016 |
| IP 22 | - | Castile and León | 2016 |
| IP 23 | Tempranillo and Tintilla | La Rioja | 2017 |
| IP 24 | Tempranillo | La Rioja | 2017 |
| IP 25 | Tempranillo and Tintilla | La Rioja | - |
| IP 26 | Graciano, Tintilla and, Tempranillo (Ecological) | La Rioja | - |
| IP 27 | - | Castile-La Mancha | 2017 |
| IP 28 | - | Castile and León | 2016 |
| IP 29 | - | Castile and León | 2016 |
| IP 30 | Tempranillo | Castile and León | 2016 |
| CV 1 | - | - | - |

| | | | |
|---|---|---|---|
| CV 2 | - | - | - |
| CV 3 | - | - | - |
| CV 4 | - | - | - |
| CV 5 | - | - | - |
| CV 6 | - | - | - |

CI: Canary Islands; IP: Iberian Peninsula; CV: Cape Verde.

**Table S4.-** Concentrations obtained of the red wine samples analysed (n = 84).

| Sample Code | Analyte concentration (mg/kg) | | | | | | | | | | |
|---|---|---|---|---|---|---|---|---|---|---|---|
| | **Azoxystrobin** | **Benalaxyl** | **Boscalid** | **Carbendazim** | **Carbofuran** | **Chlorpyrifos-methyl** | **Cyprodinil** | **Diethofencarb** | **Difenoconazole** | **Dimethomorf** | **Fenbuconazole** |
| CI 1 | n.d. | n.d. | 0.02 | n.d. | n.d. | n.d. | n.d. | n.d. | n.d. | n.d. | n.d. |
| CI 2 | n.d. | n.d. | 0.03 | 0.10 | n.d. | n.d. | n.d. | n.d. | n.d. | n.d. | n.d. |
| CI 3 | n.d. | n.d. | 0.05 | n.d. | n.d. | n.d. | < LOQ | n.d. | 0.01 | n.d. | n.d. |
| CI 4 | n.d. | n.d. | 0.02 | n.d. | n.d. | n.d. | n.d. | n.d. | n.d. | n.d. | n.d. |
| CI 5 | < LOQ | n.d. | 0.02 | 0.04 | n.d. | n.d. | n.d. | n.d. | n.d. | < LOQ | n.d. |
| CI 6 | < LOQ | n.d. | 0.01 | 0.02 | n.d. | n.d. | n.d. | n.d. | n.d. | 0.01 | n.d. |
| CI 7 | n.d. | n.d. | 0.01 | 0.06 | n.d. | n.d. | n.d. | n.d. | n.d. | < LOQ | n.d. |
| CI 8 | < LOQ | < LOQ | 0.02 | 0.05 | n.d. | < LOQ | < LOQ | n.d. | n.d. | < LOQ | n.d. |
| CI 9 | n.d. | n.d. | 0.01 | < LOQ | n.d. | n.d. | n.d. | n.d. | n.d. | n.d. | n.d. |
| CI 10 | n.d. | n.d. | 0.01 | n.d. | n.d. | n.d. | n.d. | n.d. | n.d. | n.d. | n.d. |
| CI 11 | n.d. | n.d. | 0.01 | 0.02 | n.d. | n.d. | n.d. | n.d. | n.d. | n.d. | n.d. |
| CI 12 | n.d. | n.d. | n.d. | 0.02 | n.d. | n.d. | n.d. | n.d. | n.d. | n.d. | n.d. |
| CI 13 | n.d. | n.d. | 0.03 | 0.10 | 0.004 | n.d. | n.d. | n.d. | n.d. | < LOQ | n.d. |
| CI 14 | n.d. | n.d. | < LOQ | 0.07 | n.d. | n.d. | n.d. | n.d. | n.d. | n.d. | n.d. |
| CI 15 | < LOQ | n.d. | 0.03 | < LOQ | n.d. | n.d. | < LOQ | n.d. | n.d. | 0.01 | n.d. |
| CI 16 | n.d. | n.d. | n.d. | 0.10 | n.d. | n.d. | n.d. | n.d. | n.d. | n.d. | n.d. |
| CI 17 | < LOQ | n.d. | n.d. | < LOQ | n.d. | n.d. | n.d. | n.d. | n.d. | n.d. | n.d. |
| CI 18 | n.d. | n.d. | 0.03 | 0.09 | n.d. | n.d. | 0.02 | n.d. | n.d. | 0.05 | n.d. |
| CI 19 | n.d. | n.d. | 0.03 | 0.04 | n.d. | n.d. | 0.04 | n.d. | n.d. | 0.01 | n.d. |
| CI 20 | n.d. | n.d. | n.d. | n.d. | n.d. | n.d. | n.d. | n.d. | n.d. | n.d. | n.d. |
| CI 21 | n.d. | n.d. | 0.02 | 0.02 | n.d. | n.d. | n.d. | n.d. | n.d. | n.d. | n.d. |
| CI 22 | n.d. | n.d. | 0.01 | < LOQ | n.d. | n.d. | n.d. | n.d. | n.d. | < LOQ | n.d. |
| CI 23 | n.d. | n.d. | 0.02 | 0.02 | n.d. | n.d. | n.d. | n.d. | n.d. | < LOQ | n.d. |
| CI 24 | n.d. | n.d. | n.d. | 0.05 | n.d. | n.d. | 0.05 | n.d. | n.d. | < LOQ | n.d. |
| CI 25 | < LOQ | n.d. | n.d. | 0.72 | 0.007 | n.d. | n.d. | n.d. | n.d. | n.d. | n.d. |

| Sample Code | Analyte concentration (mg/kg) | | | | | | | | | | |
|---|---|---|---|---|---|---|---|---|---|---|---|
| | Azoxystrobin | Benalaxyl | Boscalid | Carbendazim | Carbofuran | Chlorpyrifos-methyl | Cyprodinil | Diethofencarb | Difenoconazole | Dimethomorf | Fenbuconazole |
| CI 26 | n.d. | n.d. | 0.01 | 0.03 | n.d. | n.d. | < LOQ | n.d. | n.d. | n.d. | n.d. |
| CI 27 | 0.12 | n.d. | n.d. | 0.04 | n.d. | n.d. | 0.47 | n.d. | n.d. | n.d. | n.d. |
| CI 28 | n.d. | n.d. | 0.02 | 0.09 | n.d. | n.d. | n.d. | n.d. | n.d. | < LOQ | n.d. |
| CI 29 | n.d. | n.d. | < LOQ | 0.06 | n.d. | n.d. | n.d. | n.d. | n.d. | 0.01 | n.d. |
| CI 30 | n.d. | n.d. | < LOQ | 0.04 | n.d. | n.d. | n.d. | n.d. | n.d. | 0.01 | n.d. |
| CI 31 | n.d. | n.d. | n.d. | n.d. | n.d. | n.d. | n.d. | n.d. | n.d. | n.d. | n.d. |
| CI 32 | 0.05 | n.d. | < LOQ | 0.80 | n.d. | n.d. | n.d. | n.d. | n.d. | 0.01 | n.d. |
| CI 33 | n.d. | n.d. | < LOQ | 0.02 | n.d. | n.d. | n.d. | n.d. | n.d. | 0.01 | n.d. |
| CI 34 | < LOQ | n.d. | 0.08 | 0.04 | n.d. | n.d. | < LOQ | n.d. | n.d. | < LOQ | n.d. |
| CI 35 | n.d. | n.d. | 0.01 | 0.06 | n.d. | n.d. | n.d. | n.d. | n.d. | 0.02 | n.d. |
| CI 36 | n.d. | n.d. | n.d. | n.d. | n.d. | n.d. | n.d. | n.d. | n.d. | < LOQ | n.d. |
| CI 37 | < LOQ | n.d. | < LOQ | 0.03 | n.d. | n.d. | n.d. | n.d. | n.d. | < LOQ | n.d. |
| CI 38 | n.d. | n.d. | 0.02 | 0.43 | n.d. | n.d. | n.d. | n.d. | n.d. | < LOQ | n.d. |
| CI 39 | n.d. | n.d. | 0.05 | 0.02 | n.d. | n.d. | n.d. | n.d. | n.d. | 0.02 | n.d. |
| CI 40 | n.d. | n.d. | < LOQ | 0.02 | n.d. | n.d. | n.d. | n.d. | n.d. | < LOQ | n.d. |
| CI 41 | n.d. | n.d. | 0.02 | 0.03 | n.d. | n.d. | n.d. | n.d. | n.d. | n.d. | n.d. |
| CI 42 | n.d. | n.d. | < LOQ | n.d. | n.d. | n.d. | n.d. | n.d. | n.d. | n.d. | n.d. |
| CI 43 | < LOQ | n.d. | < LOQ | < LOQ | n.d. | n.d. | n.d. | n.d. | n.d. | n.d. | n.d. |
| CI 44 | n.d. | n.d. | 0.03 | 0.02 | n.d. | n.d. | n.d. | n.d. | n.d. | n.d. | n.d. |
| CI 45 | n.d. | n.d. | 0.01 | < LOQ | n.d. | n.d. | n.d. | n.d. | n.d. | n.d. | n.d. |
| CI 46 | n.d. | n.d. | < LOQ | 0.03 | n.d. | n.d. | n.d. | n.d. | n.d. | < LOQ | n.d. |
| CI 47 | < LOQ | n.d. | < LOQ | 0.35 | n.d. | n.d. | n.d. | n.d. | n.d. | n.d. | n.d. |
| CI 48 | < LOQ | n.d. | 0.02 | < LOQ | n.d. | n.d. | n.d. | n.d. | n.d. | n.d. | n.d. |
| IP 1 | < LOQ | n.d. | 0.01 | 0.02 | n.d. | n.d. | n.d. | n.d. | n.d. | < LOQ | n.d. |
| IP 2 | n.d. | n.d. | < LOQ | n.d. | n.d. | n.d. | n.d. | n.d. | n.d. | n.d. | n.d. |
| IP 3 | < LOQ | n.d. | < LOQ | 0.02 | n.d. | n.d. | n.d. | n.d. | n.d. | < LOQ | n.d. |

| Sample Code | Analyte concentration (mg/kg) | | | | | | | | | | |
|---|---|---|---|---|---|---|---|---|---|---|---|
| | **Azoxystrobin** | **Benalaxyl** | **Boscalid** | **Carbendazim** | **Carbofuran** | **Chlorpyrifos-methyl** | **Cyprodinil** | **Diethofencarb** | **Difenoconazole** | **Dimethomorf** | **Fenbuconazole** |
| IP 4 | n.d. | n.d. | 0.01 | < LOQ | n.d. | n.d. | < LOQ | n.d. | n.d. | 0.01 | n.d. |
| IP 5 | < LOQ | n.d. | 0.01 | < LOQ | n.d. | n.d. | < LOQ | n.d. | n.d. | 0.01 | n.d. |
| IP 6 | n.d. | n.d. | < LOQ | n.d. | n.d. | n.d. | n.d. | n.d. | n.d. | n.d. | n.d. |
| IP 7 | n.d. | n.d. | 0.01 | n.d. | n.d. | n.d. | n.d. | n.d. | n.d. | n.d. | n.d. |
| IP 8 | < LOQ | n.d. | 0.02 | < LOQ | n.d. | n.d. | n.d. | n.d. | n.d. | < LOQ | n.d. |
| IP 9 | < LOQ | n.d. | n.d. | n.d. | n.d. | n.d. | n.d. | n.d. | n.d. | n.d. | n.d. |
| IP 10 | n.d. | n.d. | 0.02 | 0.01 | n.d. | n.d. | n.d. | n.d. | n.d. | 0.04 | n.d. |
| IP 11 | < LOQ | n.d. | 0.01 | 0.03 | n.d. | n.d. | < LOQ | n.d. | n.d. | < LOQ | n.d. |
| IP 12 | n.d. | n.d. | n.d. | n.d. | n.d. | n.d. | n.d. | n.d. | n.d. | n.d. | n.d. |
| IP 13 | n.d. | n.d. | n.d. | n.d. | n.d. | n.d. | n.d. | n.d. | n.d. | n.d. | n.d. |
| IP 14 | < LOQ | n.d. | 0.02 | < LOQ | n.d. | n.d. | n.d. | n.d. | n.d. | 0.01 | n.d. |
| IP 15 | n.d. | n.d. | 0.01 | 0.02 | n.d. | n.d. | n.d. | n.d. | n.d. | < LOQ | n.d. |
| IP 16 | < LOQ | n.d. | < LOQ | < LOQ | n.d. | n.d. | n.d. | n.d. | n.d. | 0.01 | n.d. |
| IP 17 | n.d. | n.d. | < LOQ | n.d. | n.d. | n.d. | n.d. | n.d. | n.d. | < LOQ | n.d. |
| IP 18 | n.d. | n.d. | n.d. | n.d. | n.d. | n.d. | n.d. | n.d. | n.d. | n.d. | n.d. |
| IP 19 | n.d. | n.d. | n.d. | n.d. | n.d. | n.d. | n.d. | n.d. | n.d. | < LOQ | n.d. |
| IP 20 | n.d. | n.d. | n.d. | n.d. | n.d. | n.d. | n.d. | n.d. | n.d. | n.d. | n.d. |
| IP 21 | n.d. | n.d. | < LOQ | < LOQ | n.d. | n.d. | n.d. | n.d. | n.d. | 0.01 | n.d. |
| IP 22 | n.d. | < LOQ | n.d. | 0.38 | n.d. | n.d. | < LOQ | < LOQ | n.d. | n.d. | n.d. |
| IP 23 | n.d. | n.d. | 0.01 | < LOQ | n.d. | n.d. | < LOQ | n.d. | n.d. | < LOQ | n.d. |
| IP 24 | n.d. | n.d. | 0.02 | < LOQ | n.d. | n.d. | n.d. | n.d. | n.d. | 0.01 | n.d. |
| IP 25 | < LOQ | n.d. | 0.01 | < LOQ | n.d. | n.d. | < LOQ | n.d. | n.d. | < LOQ | n.d. |
| IP 26 | n.d. | n.d. | n.d. | n.d. | n.d. | n.d. | n.d. | n.d. | n.d. | n.d. | n.d. |
| IP 27 | n.d. | n.d. | < LOQ | n.d. | n.d. | n.d. | n.d. | n.d. | n.d. | n.d. | < LOQ |
| IP 28 | n.d. | n.d. | n.d. | 0.16 | n.d. | n.d. | n.d. | n.d. | n.d. | n.d. | n.d. |
| IP 29 | n.d. | n.d. | < LOQ | < LOQ | n.d. | n.d. | n.d. | n.d. | n.d. | < LOQ | n.d. |

| Sample Code | Analyte concentration (mg/kg) | | | | | | | | | | |
|---|---|---|---|---|---|---|---|---|---|---|---|
| | **Azoxystrobin** | **Benalaxyl** | **Boscalid** | **Carbendazim** | **Carbofuran** | **Chlorpyrifos-methyl** | **Cyprodinil** | **Diethofencarb** | **Difenoconazole** | **Dimethomorf** | **Fenbuconazole** |
| IP 30 | n.d. | n.d. | n.d. | n.d. | n.d. | n.d. | n.d. | n.d. | n.d. | n.d. | n.d. |
| CV 1 | n.d. | n.d. | n.d. | n.d. | n.d. | n.d. | n.d. | n.d. | n.d. | n.d. | n.d. |
| CV 2 | n.d. | n.d. | n.d. | n.d. | n.d. | n.d. | n.d. | n.d. | n.d. | n.d. | n.d. |
| CV 3 | n.d. | n.d. | n.d. | n.d. | n.d. | n.d. | n.d. | n.d. | n.d. | < LOQ | n.d. |
| CV 4 | n.d. | n.d. | n.d. | n.d. | n.d. | n.d. | n.d. | n.d. | n.d. | n.d. | n.d. |
| CV 5 | n.d. | n.d. | n.d. | n.d. | n.d. | n.d. | n.d. | n.d. | n.d. | n.d. | n.d. |
| CV 6 | n.d. | n.d. | n.d. | n.d. | n.d. | n.d. | n.d. | n.d. | n.d. | n.d. | n.d. |

| Sample Code | Analyte concentration (mg/kg) | | | | | | | | | |
|---|---|---|---|---|---|---|---|---|---|---|
| | **Fenhexamide** | **Fludioxonil** | **Imidacloprid** | **Iprodione** | **Iprovalicarb** | **Kresoxim-methyl** | **Metalaxyl** | **Methoxyfenozide** | **Metrafenone** | **Myclobutanil** |
| CI 1 | n.d. | n.d. | n.d. | 0.01 | n.d. | n.d. | 0.04 | n.d. | n.d. | < LOQ |
| CI 2 | 0.01 | n.d. | n.d. | 0.09 | n.d. | < LOQ | 0.09 | n.d. | n.d. | < LOQ |
| CI 3 | n.d. | n.d. | n.d. | 0.02 | < LOQ | n.d. | 0.13 | n.d. | < LOQ | n.d. |
| CI 4 | n.d. | n.d. | n.d. | < LOQ | n.d. | < LOQ | n.d. | 0.01 | n.d. | < LOQ |
| CI 5 | n.d. | n.d. | 0.02 | 0.02 | n.d. | n.d. | < LOQ | n.d. | n.d. | 0.02 |
| CI 6 | n.d. | n.d. | n.d. | < LOQ | n.d. | n.d. | n.d. | n.d. | 0.01 | n.d. |
| CI 7 | n.d. | n.d. | n.d. | 0.40 | n.d. | < LOQ | 0.41 | n.d. | < LOQ | < LOQ |
| CI 8 | 0.11 | n.d. | n.d. | 0.08 | < LOQ | n.d. | 0.03 | n.d. | n.d. | < LOQ |
| CI 9 | n.d. | n.d. | n.d. | 0.18 | n.d. | n.d. | 0.11 | n.d. | n.d. | < LOQ |
| CI 10 | n.d. | n.d. | n.d. | 0.01 | n.d. | n.d. | 0.01 | n.d. | n.d. | n.d. |
| CI 11 | n.d. | n.d. | n.d. | 0.16 | n.d. | n.d. | 0.03 | n.d. | n.d. | n.d. |
| CI 12 | n.d. | n.d. | n.d. | 0.06 | n.d. | n.d. | n.d. | n.d. | n.d. | n.d. |
| CI 13 | 0.11 | n.d. | n.d. | 0.20 | n.d. | < LOQ | 0.09 | n.d. | n.d. | < LOQ |
| CI 14 | 0.13 | n.d. | 0.02 | 0.16 | n.d. | n.d. | n.d. | n.d. | < LOQ | n.d. |
| CI 15 | n.d. | < LOQ | n.d. | 0.03 | n.d. | n.d. | n.d. | n.d. | < LOQ | n.d. |

| Sample Code | Analyte concentration (mg/kg) | | | | | | | | | |
|---|---|---|---|---|---|---|---|---|---|---|
| | Fenhexamide | Fludioxonil | Imidacloprid | Iprodione | Iprovalicarb | Kresoxim-methyl | Metalaxyl | Methoxyfenozide | Metrafenone | Myclobutanil |
| CI 16 | n.d. | n.d. | n.d. | 0.10 | n.d. | n.d. | < LOQ | n.d. | n.d. | n.d. |
| CI 17 | n.d. | n.d. | n.d. | n.d. | n.d. | n.d. | < LOQ | n.d. | n.d. | n.d. |
| CI 18 | n.d. | n.d. | < LOQ | 0.11 | n.d. | < LOQ | 0.10 | n.d. | n.d. | n.d. |
| CI 19 | n.d. | n.d. | n.d. | 0.20 | n.d. | n.d. | 0.18 | n.d. | n.d. | < LOQ |
| CI 20 | n.d. | n.d. | n.d. | n.d. | n.d. | n.d. | n.d. | n.d. | n.d. | n.d. |
| CI 21 | n.d. | n.d. | n.d. | n.d. | n.d. | n.d. | 0.05 | n.d. | n.d. | n.d. |
| CI 22 | n.d. | n.d. | n.d. | n.d. | n.d. | n.d. | n.d. | n.d. | n.d. | n.d. |
| CI 23 | n.d. | n.d. | n.d. | 0.01 | n.d. | n.d. | n.d. | n.d. | n.d. | n.d. |
| CI 24 | n.d. | n.d. | n.d. | 0.03 | n.d. | n.d. | 0.01 | n.d. | n.d. | n.d. |
| CI 25 | n.d. | n.d. | n.d. | n.d. | n.d. | n.d. | 0.09 | n.d. | n.d. | n.d. |
| CI 26 | < LOQ | n.d. | n.d. | 0.02 | n.d. | < LOQ | 0.02 | n.d. | < LOQ | n.d. |
| CI 27 | 0.05 | n.d. | n.d. | n.d. | n.d. | n.d. | 0.06 | n.d. | n.d. | n.d. |
| CI 28 | 0.02 | n.d. | n.d. | 0.03 | n.d. | < LOQ | 0.05 | n.d. | n.d. | n.d. |
| CI 29 | n.d. | n.d. | 0.02 | 0.01 | n.d. | n.d. | 0.02 | n.d. | n.d. | n.d. |
| CI 30 | n.d. | n.d. | n.d. | 0.09 | n.d. | n.d. | 0.32 | n.d. | < LOQ | n.d. |
| CI 31 | n.d. | n.d. | n.d. | n.d. | n.d. | n.d. | n.d. | n.d. | n.d. | n.d. |
| CI 32 | n.d. | n.d. | < LOQ | 0.02 | n.d. | < LOQ | 0.02 | n.d. | n.d. | n.d. |
| CI 33 | < LOQ | n.d. | n.d. | 0.03 | n.d. | n.d. | 0.04 | n.d. | n.d. | < LOQ |
| CI 34 | 0.02 | n.d. | n.d. | 0.04 | n.d. | n.d. | 0.02 | n.d. | n.d. | < LOQ |
| CI 35 | n.d. | n.d. | n.d. | 0.04 | n.d. | n.d. | 0.01 | n.d. | n.d. | n.d. |
| CI 36 | n.d. | n.d. | n.d. | n.d. | n.d. | n.d. | < LOQ | n.d. | n.d. | n.d. |
| CI 37 | n.d. | n.d. | 0.02 | 0.02 | n.d. | n.d. | n.d. | n.d. | n.d. | < LOQ |
| CI 38 | n.d. | n.d. | < LOQ | 0.11 | n.d. | n.d. | 0.02 | n.d. | n.d. | n.d. |
| CI 39 | n.d. | n.d. | < LOQ | 0.37 | n.d. | 0.01 | < LOQ | n.d. | < LOQ | n.d. |
| CI 40 | n.d. | n.d. | n.d. | < LOQ | n.d. | n.d. | < LOQ | n.d. | n.d. | n.d. |

| Sample Code | Analyte concentration (mg/kg) | | | | | | | | | |
|---|---|---|---|---|---|---|---|---|---|---|
| | Fenhexamide | Fludioxonil | Imidacloprid | Iprodione | Iprovalicarb | Kresoxim-methyl | Metalaxyl | Methoxyfenozide | Metrafenone | Myclobutanil |
| CI 41 | 0.01 | n.d. | n.d. | 0.13 | n.d. | n.d. | 0.01 | n.d. | n.d. | < LOQ |
| CI 42 | n.d. | n.d. | n.d. | 0.04 | n.d. | n.d. | n.d. | n.d. | n.d. | n.d. |
| CI 43 | n.d. | n.d. | n.d. | 0.05 | n.d. | n.d. | < LOQ | n.d. | n.d. | < LOQ |
| CI 44 | 0.06 | n.d. | n.d. | 0.01 | n.d. | n.d. | 0.11 | n.d. | n.d. | n.d. |
| CI 45 | n.d. | n.d. | n.d. | 0.02 | n.d. | n.d. | n.d. | n.d. | n.d. | n.d. |
| CI 46 | n.d. | n.d. | n.d. | 0.01 | n.d. | < LOQ | 0.05 | n.d. | < LOQ | n.d. |
| CI 47 | n.d. | n.d. | n.d. | n.d. | n.d. | n.d. | 0.11 | n.d. | n.d. | n.d. |
| CI 48 | n.d. | n.d. | n.d. | n.d. | n.d. | n.d. | 0.02 | n.d. | n.d. | n.d. |
| IP 1 | n.d. | n.d. | n.d. | n.d. | n.d. | < LOQ | n.d. | n.d. | n.d. | < LOQ |
| IP 2 | n.d. | n.d. | n.d. | n.d. | n.d. | n.d. | < LOQ | n.d. | n.d. | n.d. |
| IP 3 | n.d. | < LOQ | n.d. | n.d. | n.d. | n.d. | n.d. | n.d. | n.d. | < LOQ |
| IP 4 | n.d. | < LOQ | < LOQ | n.d. | n.d. | n.d. | 0.03 | 0.02 | n.d. | < LOQ |
| IP 5 | n.d. | < LOQ | < LOQ | n.d. | n.d. | n.d. | 0.03 | 0.01 | n.d. | < LOQ |
| IP 6 | n.d. | n.d. | n.d. | < LOQ | n.d. | n.d. | < LOQ | n.d. | n.d. | n.d. |
| IP 7 | n.d. | n.d. | n.d. | n.d. | n.d. | n.d. | n.d. | n.d. | n.d. | n.d. |
| IP 8 | n.d. | < LOQ | < LOQ | n.d. | n.d. | n.d. | 0.03 | n.d. | < LOQ | n.d. |
| IP 9 | n.d. | 0.02 | n.d. | n.d. | n.d. | n.d. | n.d. | 0.13 | n.d. | n.d. |
| IP 10 | n.d. | n.d. | 0.02 | n.d. | < LOQ | n.d. | 0.02 | n.d. | n.d. | < LOQ |
| IP 11 | n.d. | < LOQ | n.d. | n.d. | n.d. | n.d. | n.d. | n.d. | n.d. | n.d. |
| IP 12 | n.d. | n.d. | 0.02 | n.d. | n.d. | n.d. | n.d. | n.d. | n.d. | n.d. |
| IP 13 | n.d. | n.d. | 0.02 | < LOQ | n.d. | n.d. | n.d. | n.d. | n.d. | n.d. |
| IP 14 | n.d. | n.d. | n.d. | 0.03 | n.d. | n.d. | 0.02 | n.d. | n.d. | n.d. |
| IP 15 | n.d. | n.d. | n.d. | n.d. | n.d. | n.d. | n.d. | n.d. | n.d. | n.d. |
| IP 16 | n.d. | n.d. | n.d. | n.d. | n.d. | n.d. | 0.03 | n.d. | n.d. | n.d. |
| IP 17 | n.d. | n.d. | 0.03 | n.d. | n.d. | n.d. | n.d. | n.d. | n.d. | n.d. |

| Sample Code | Analyte concentration (mg/kg) | | | | | | | | | |
|---|---|---|---|---|---|---|---|---|---|---|
| | Fenhexamide | Fludioxonil | Imidacloprid | Iprodione | Iprovalicarb | Kresoxim-methyl | Metalaxyl | Methoxyfenozide | Metrafenone | Myclobutanil |
| IP 18 | n.d. | n.d. | n.d. | n.d. | n.d. | n.d. | n.d. | n.d. | n.d. | n.d. |
| IP 19 | n.d. | n.d. | n.d. | n.d. | n.d. | n.d. | n.d. | n.d. | n.d. | n.d. |
| IP 20 | n.d. | n.d. | n.d. | n.d. | < LOQ | n.d. | n.d. | n.d. | n.d. | n.d. |
| IP 21 | n.d. | n.d. | n.d. | n.d. | n.d. | n.d. | 0.02 | n.d. | n.d. | n.d. |
| IP 22 | n.d. | n.d. | n.d. | n.d. | n.d. | n.d. | n.d. | n.d. | n.d. | n.d. |
| IP 23 | n.d. | n.d. | < LOQ | n.d. | n.d. | n.d. | 0.02 | n.d. | n.d. | n.d. |
| IP 24 | n.d. | n.d. | n.d. | n.d. | n.d. | n.d. | 0.05 | 0.01 | n.d. | < LOQ |
| IP 25 | n.d. | n.d. | < LOQ | n.d. | n.d. | n.d. | 0.04 | 0.01 | n.d. | n.d. |
| IP 26 | n.d. | n.d. | n.d. | n.d. | n.d. | < LOQ | n.d. | n.d. | n.d. | n.d. |
| IP 27 | n.d. | n.d. | n.d. | n.d. | n.d. | n.d. | n.d. | n.d. | n.d. | < LOQ |
| IP 28 | n.d. | n.d. | n.d. | n.d. | n.d. | n.d. | 0.01 | n.d. | n.d. | n.d. |
| IP 29 | 0.02 | < LOQ | n.d. | < LOQ | n.d. | n.d. | n.d. | n.d. | n.d. | < LOQ |
| IP 30 | n.d. | n.d. | n.d. | n.d. | n.d. | n.d. | < LOQ | n.d. | n.d. | n.d. |
| CV 1 | n.d. | n.d. | n.d. | n.d. | n.d. | < LOQ | n.d. | n.d. | n.d. | n.d. |
| CV 2 | n.d. | n.d. | n.d. | n.d. | n.d. | 0.02 | n.d. | n.d. | n.d. | n.d. |
| CV 3 | n.d. | n.d. | n.d. | n.d. | n.d. | < LOQ | n.d. | n.d. | n.d. | n.d. |
| CV 4 | n.d. | n.d. | n.d. | n.d. | n.d. | < LOQ | n.d. | n.d. | n.d. | n.d. |
| CV 5 | n.d. | n.d. | n.d. | n.d. | n.d. | < LOQ | n.d. | n.d. | n.d. | n.d. |
| CV 6 | n.d. | n.d. | n.d. | n.d. | n.d. | 0.08 | n.d. | n.d. | n.d. | n.d. |

| Sample Code | Analyte concentration (mg/kg) | | | | | | | | | |
|---|---|---|---|---|---|---|---|---|---|---|
| | Nuarimol | Oxadixyl | Penconazole | Pyraclostrobin | Pyrimethanil | Tebuconazole | Tetraconazole | Thiophanate-methyl | Triadimefon | Triadimenol |
| CI 1 | n.d. | n.d. | n.d. | n.d. | n.d. | n.d. | n.d. | n.d. | n.d. | n.d. |
| CI 2 | n.d. | n.d. | n.d. | n.d. | 0.40 | < LOQ | n.d. | 0.06 | n.d. | n.d. |
| CI 3 | n.d. | n.d. | n.d. | n.d. | n.d. | 0.01 | n.d. | < LOQ | n.d. | n.d. |

| Sample Code | Analyte concentration (mg/kg) | | | | | | | | | |
|---|---|---|---|---|---|---|---|---|---|---|
| | **Nuarimol** | **Oxadixyl** | **Penconazole** | **Pyraclostrobin** | **Pyrimethanil** | **Tebuconazole** | **Tetraconazole** | **Thiophanate-methyl** | **Triadimefon** | **Triadimenol** |
| CI 4 | n.d. | n.d. | n.d. | n.d. | 0.01 | < LOQ | n.d. | n.d. | n.d. | 0.02 |
| CI 5 | n.d. | n.d. | n.d. | n.d. | < LOQ | 0.02 | n.d. | < LOQ | n.d. | n.d. |
| CI 6 | n.d. | n.d. | n.d. | n.d. | 0.04 | 0.27 | < LOQ | < LOQ | n.d. | n.d. |
| CI 7 | n.d. | n.d. | n.d. | 0.02 | 0.05 | 0.02 | 0.02 | n.d. | n.d. | n.d. |
| CI 8 | n.d. | n.d. | n.d. | n.d. | 0.04 | 0.02 | n.d. | < LOQ | n.d. | n.d. |
| CI 9 | n.d. | n.d. | n.d. | n.d. | 0.03 | 0.04 | < LOQ | n.d. | n.d. | n.d. |
| CI 10 | n.d. | n.d. | n.d. | n.d. | n.d. | 0.05 | n.d. | n.d. | n.d. | n.d. |
| CI 11 | n.d. | n.d. | n.d. | n.d. | 0.05 | < LOQ | n.d. | < LOQ | n.d. | n.d. |
| CI 12 | n.d. | n.d. | n.d. | n.d. | 0.05 | n.d. | n.d. | n.d. | n.d. | n.d. |
| CI 13 | n.d. | n.d. | n.d. | n.d. | 0.05 | 0.03 | < LOQ | 0.07 | n.d. | n.d. |
| CI 14 | n.d. | n.d. | n.d. | n.d. | 0.02 | 0.02 | n.d. | 0.10 | n.d. | 0.03 |
| CI 15 | n.d. | n.d. | n.d. | n.d. | 0.01 | n.d. | n.d. | n.d. | n.d. | n.d. |
| CI 16 | n.d. | n.d. | n.d. | n.d. | 0.02 | 0.01 | < LOQ | 0.04 | n.d. | 0.05 |
| CI 17 | n.d. | n.d. | n.d. | n.d. | n.d. | n.d. | n.d. | n.d. | n.d. | n.d. |
| CI 18 | n.d. | n.d. | n.d. | n.d. | 0.05 | 0.01 | < LOQ | 0.02 | n.d. | n.d. |
| CI 19 | n.d. | n.d. | n.d. | n.d. | 0.15 | 0.01 | < LOQ | < LOQ | n.d. | n.d. |
| CI 20 | n.d. | n.d. | n.d. | n.d. | n.d. | n.d. | n.d. | n.d. | n.d. | n.d. |
| CI 21 | n.d. | n.d. | n.d. | n.d. | n.d. | < LOQ | n.d. | < LOQ | n.d. | n.d. |
| CI 22 | n.d. | n.d. | n.d. | n.d. | n.d. | 0.01 | n.d. | n.d. | n.d. | n.d. |
| CI 23 | n.d. | n.d. | n.d. | n.d. | 0.07 | 0.03 | n.d. | < LOQ | n.d. | n.d. |
| CI 24 | n.d. | n.d. | < LOQ | n.d. | 0.07 | < LOQ | < LOQ | 0.02 | n.d. | n.d. |
| CI 25 | < LOQ | 0.02 | < LOQ | n.d. | n.d. | n.d. | n.d. | n.d. | n.d. | n.d. |
| CI 26 | n.d. | n.d. | n.d. | n.d. | 0.02 | < LOQ | n.d. | 0.02 | n.d. | n.d. |
| CI 27 | n.d. | n.d. | 0.01 | n.d. | < LOQ | < LOQ | n.d. | 0.02 | n.d. | n.d. |
| CI 28 | n.d. | n.d. | n.d. | n.d. | 0.06 | < LOQ | < LOQ | 0.10 | n.d. | n.d. |

| Sample Code | Analyte concentration (mg/kg) | | | | | | | | | |
|---|---|---|---|---|---|---|---|---|---|---|
| | Nuarimol | Oxadixyl | Penconazole | Pyraclostrobin | Pyrimethanil | Tebuconazole | Tetraconazole | Thiophanate-methyl | Triadimefon | Triadimenol |
| CI 29 | n.d. | n.d. | n.d. | n.d. | n.d. | 0.04 | < LOQ | 0.06 | n.d. | 0.02 |
| CI 30 | n.d. | n.d. | n.d. | n.d. | n.d. | < LOQ | 0.02 | 0.03 | n.d. | < LOQ |
| CI 31 | n.d. | n.d. | n.d. | n.d. | n.d. | n.d. | n.d. | n.d. | n.d. | n.d. |
| CI 32 | n.d. | n.d. | n.d. | n.d. | 0.02 | 0.01 | n.d. | 1.29 | n.d. | n.d. |
| CI 33 | n.d. | n.d. | n.d. | n.d. | 0.02 | < LOQ | n.d. | 0.02 | n.d. | n.d. |
| CI 34 | n.d. | n.d. | n.d. | n.d. | 0.01 | 0.01 | n.d. | < LOQ | n.d. | n.d. |
| CI 35 | n.d. | n.d. | n.d. | n.d. | 0.01 | < LOQ | n.d. | 0.03 | n.d. | n.d. |
| CI 36 | n.d. | n.d. | n.d. | n.d. | n.d. | < LOQ | n.d. | n.d. | n.d. | n.d. |
| CI 37 | n.d. | n.d. | n.d. | n.d. | n.d. | < LOQ | n.d. | 0.02 | n.d. | < LOQ |
| CI 38 | n.d. | n.d. | n.d. | n.d. | 0.03 | n.d. | n.d. | 0.55 | n.d. | n.d. |
| CI 39 | n.d. | n.d. | n.d. | n.d. | 0.04 | < LOQ | n.d. | < LOQ | n.d. | n.d. |
| CI 40 | n.d. | n.d. | n.d. | n.d. | 0.02 | 0.01 | n.d. | < LOQ | n.d. | n.d. |
| CI 41 | n.d. | n.d. | < LOQ | n.d. | 0.61 | 0.03 | < LOQ | 0.02 | n.d. | n.d. |
| CI 42 | n.d. | n.d. | n.d. | n.d. | n.d. | n.d. | n.d. | n.d. | n.d. | n.d. |
| CI 43 | n.d. | n.d. | n.d. | n.d. | 0.07 | < LOQ | n.d. | 0.02 | n.d. | n.d. |
| CI 44 | n.d. | n.d. | n.d. | n.d. | 0.02 | n.d. | n.d. | n.d. | n.d. | n.d. |
| CI 45 | n.d. | n.d. | n.d. | n.d. | < LOQ | n.d. | n.d. | n.d. | n.d. | n.d. |
| CI 46 | n.d. | n.d. | n.d. | n.d. | 0.03 | 0.01 | n.d. | 0.05 | n.d. | n.d. |
| CI 47 | n.d. | n.d. | n.d. | n.d. | 0.02 | < LOQ | n.d. | 0.15 | n.d. | n.d. |
| CI 48 | n.d. | n.d. | n.d. | n.d. | 0.03 | < LOQ | n.d. | n.d. | n.d. | n.d. |
| IP 1 | n.d. | n.d. | n.d. | n.d. | n.d. | 0.01 | n.d. | 0.03 | n.d. | n.d. |
| IP 2 | n.d. | n.d. | n.d. | n.d. | n.d. | < LOQ | n.d. | n.d. | n.d. | n.d. |
| IP 3 | n.d. | n.d. | n.d. | n.d. | n.d. | 0.01 | n.d. | < LOQ | n.d. | n.d. |
| IP 4 | n.d. | n.d. | n.d. | n.d. | n.d. | 0.01 | n.d. | n.d. | n.d. | n.d. |
| IP 5 | n.d. | n.d. | n.d. | n.d. | n.d. | < LOQ | n.d. | n.d. | n.d. | n.d. |

| Sample Code | Analyte concentration (mg/kg) | | | | | | | | | |
|---|---|---|---|---|---|---|---|---|---|---|
| | Nuarimol | Oxadixyl | Penconazole | Pyraclostrobin | Pyrimethanil | Tebuconazole | Tetraconazole | Thiophanate-methyl | Triadimefon | Triadimenol |
| IP 6 | n.d. | n.d. | n.d. | n.d. | n.d. | < LOQ | n.d. | n.d. | n.d. | n.d. |
| IP 7 | n.d. | n.d. | n.d. | n.d. | n.d. | < LOQ | n.d. | n.d. | n.d. | n.d. |
| IP 8 | n.d. | n.d. | n.d. | n.d. | n.d. | < LOQ | n.d. | < LOQ | n.d. | 0.02 |
| IP 9 | n.d. | n.d. | n.d. | n.d. | n.d. | n.d. | n.d. | n.d. | < LOQ | n.d. |
| IP 10 | n.d. | n.d. | n.d. | n.d. | n.d. | n.d. | n.d. | < LOQ | n.d. | n.d. |
| IP 11 | n.d. | n.d. | n.d. | n.d. | n.d. | 0.01 | n.d. | < LOQ | n.d. | n.d. |
| IP 12 | n.d. | n.d. | n.d. | n.d. | n.d. | n.d. | n.d. | n.d. | n.d. | n.d. |
| IP 13 | n.d. | n.d. | n.d. | n.d. | n.d. | 0.03 | < LOQ | n.d. | n.d. | n.d. |
| IP 14 | n.d. | n.d. | n.d. | n.d. | 0.04 | < LOQ | n.d. | n.d. | n.d. | n.d. |
| IP 15 | n.d. | n.d. | n.d. | n.d. | n.d. | 0.02 | n.d. | < LOQ | n.d. | n.d. |
| IP 16 | n.d. | n.d. | n.d. | n.d. | < LOQ | < LOQ | n.d. | n.d. | n.d. | n.d. |
| IP 17 | n.d. | n.d. | n.d. | n.d. | n.d. | < LOQ | n.d. | n.d. | n.d. | n.d. |
| IP 18 | n.d. | n.d. | n.d. | n.d. | n.d. | n.d. | n.d. | n.d. | n.d. | n.d. |
| IP 19 | n.d. | n.d. | n.d. | n.d. | n.d. | n.d. | n.d. | n.d. | n.d. | n.d. |
| IP 20 | n.d. | n.d. | n.d. | n.d. | n.d. | n.d. | n.d. | n.d. | n.d. | n.d. |
| IP 21 | n.d. | n.d. | n.d. | n.d. | < LOQ | < LOQ | n.d. | n.d. | n.d. | n.d. |
| IP 22 | n.d. | n.d. | n.d. | n.d. | < LOQ | < LOQ | n.d. | 0.12 | n.d. | n.d. |
| IP 23 | n.d. | n.d. | n.d. | n.d. | < LOQ | < LOQ | n.d. | n.d. | n.d. | n.d. |
| IP 24 | n.d. | n.d. | n.d. | n.d. | n.d. | < LOQ | n.d. | n.d. | n.d. | n.d. |
| IP 25 | n.d. | n.d. | n.d. | n.d. | n.d. | n.d. | n.d. | < LOQ | n.d. | n.d. |
| IP 26 | n.d. | n.d. | n.d. | n.d. | n.d. | n.d. | n.d. | n.d. | n.d. | n.d. |
| IP 27 | n.d. | n.d. | n.d. | n.d. | n.d. | n.d. | n.d. | n.d. | n.d. | n.d. |
| IP 28 | n.d. | n.d. | n.d. | n.d. | n.d. | 0.03 | < LOQ | 0.12 | n.d. | n.d. |
| IP 29 | n.d. | n.d. | n.d. | n.d. | n.d. | 0.01 | n.d. | < LOQ | n.d. | n.d. |
| IP 30 | n.d. | n.d. | n.d. | n.d. | n.d. | < LOQ | n.d. | n.d. | n.d. | n.d. |

| Sample Code | Analyte concentration (mg/kg) | | | | | | | | | |
|---|---|---|---|---|---|---|---|---|---|---|
| | **Nuarimol** | **Oxadixyl** | **Penconazole** | **Pyraclostrobin** | **Pyrimethanil** | **Tebuconazole** | **Tetraconazole** | **Thiophanate-methyl** | **Triadimefon** | **Triadimenol** |
| CV 1 | n.d. | n.d. | n.d. | n.d. | n.d. | < LOQ | n.d. | n.d. | n.d. | n.d. |
| CV 2 | n.d. | n.d. | n.d. | n.d. | n.d. | 0.04 | n.d. | n.d. | n.d. | n.d. |
| CV 3 | n.d. | n.d. | n.d. | n.d. | n.d. | < LOQ | n.d. | n.d. | n.d. | n.d. |
| CV 4 | n.d. | n.d. | n.d. | n.d. | n.d. | < LOQ | n.d. | n.d. | n.d. | n.d. |
| CV 5 | n.d. | n.d. | n.d. | n.d. | n.d. | < LOQ | n.d. | n.d. | n.d. | n.d. |
| CV 6 | n.d. | n.d. | n.d. | n.d. | n.d. | n.d. | n.d. | n.d. | n.d. | n.d. |

CI: Canary Islands; CV: Cape Verde; IP: Iberian Peninsula; LOQ: limit of quantification; n.d.: not detected.

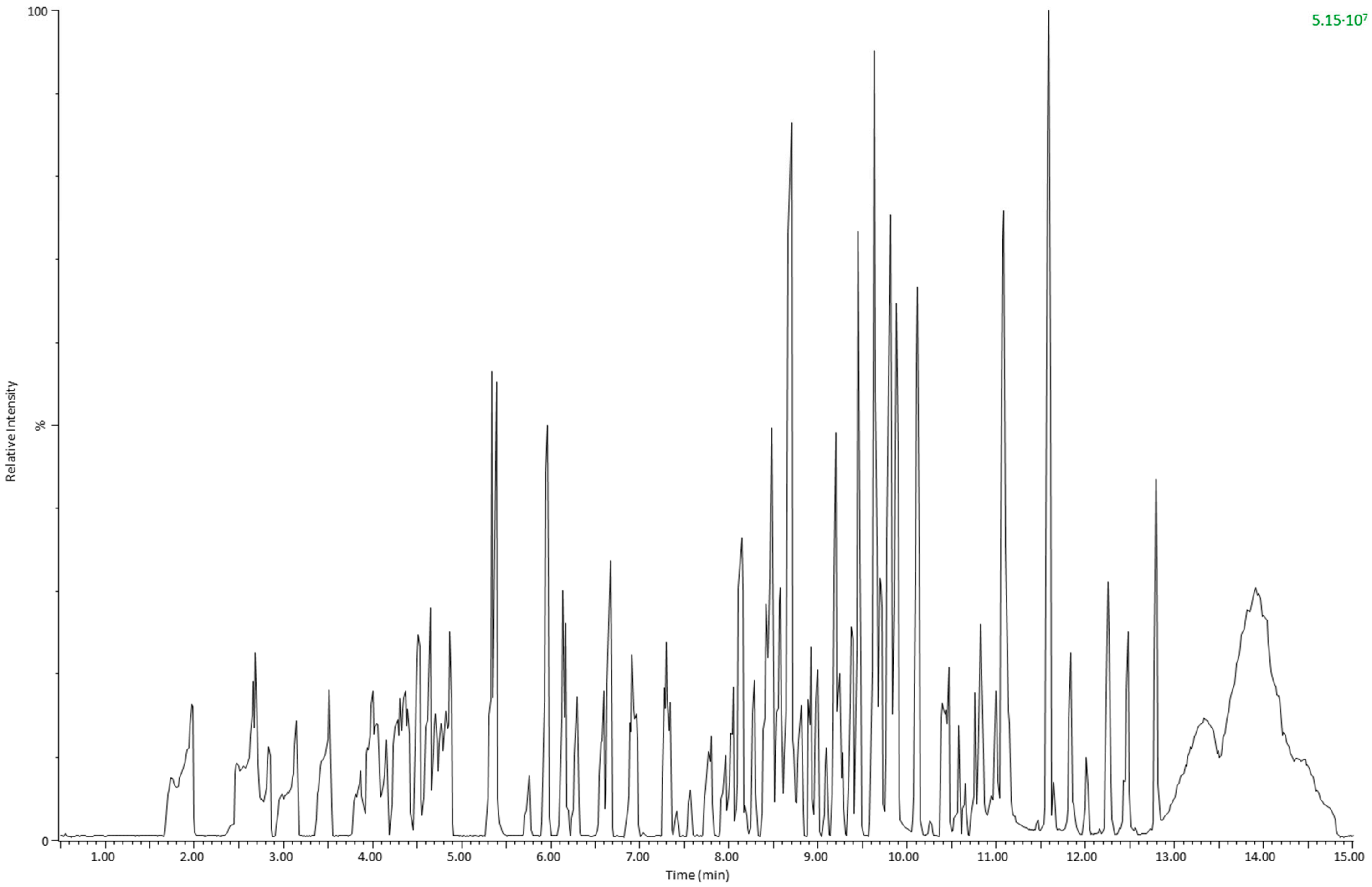


Figure S1. UHPLC-MSFull scan chromatogram of a standard solution spiked with the pesticides and TPP at a concentration level of 100 µg/L.

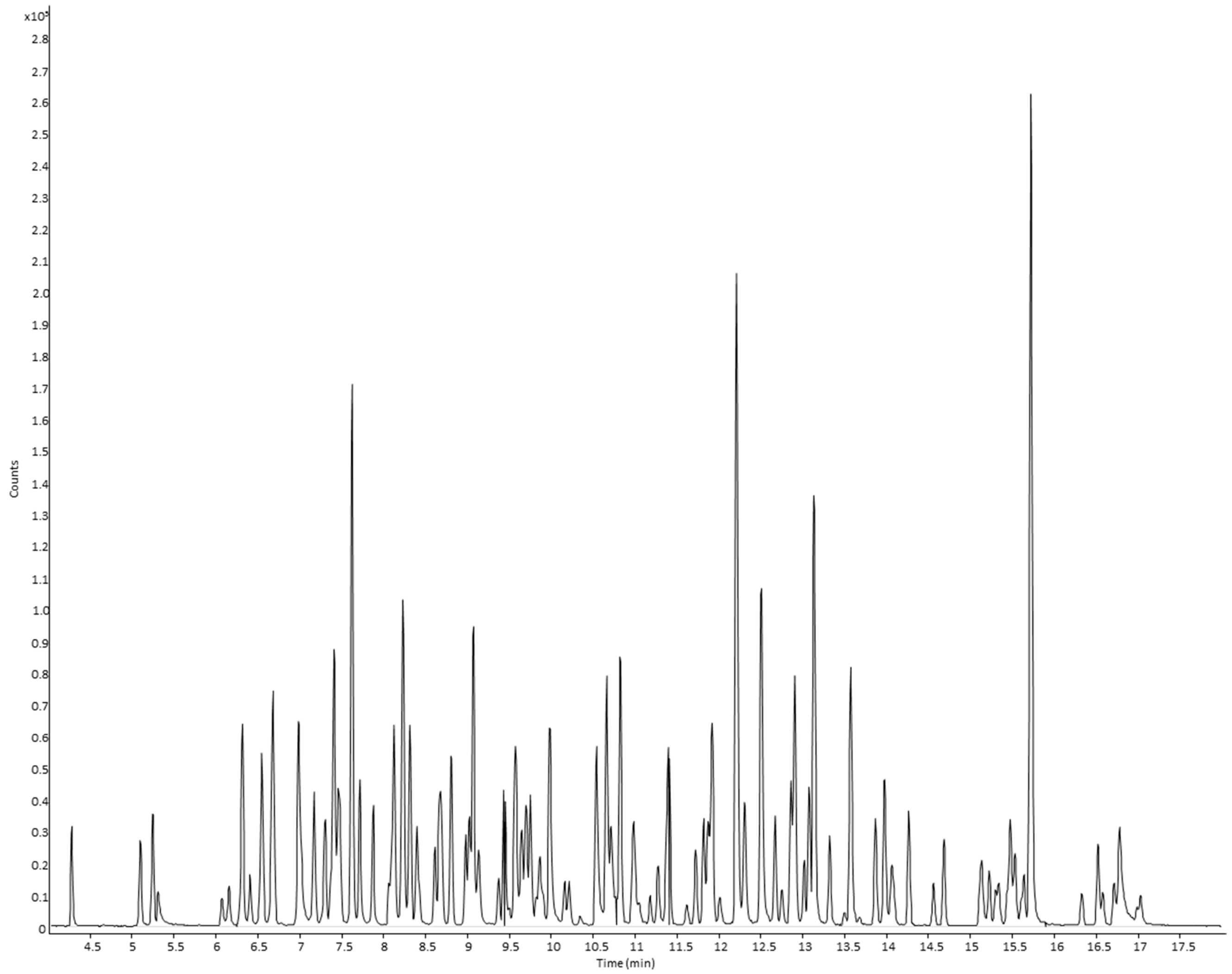


Figure S2. GC-MS total ion chromatogram of a standard solution spiked with the pesticides and TPP at a concentration level of 100 µg/L.
